# Spinal motoneuron excitability is homeostatically regulated through β-adrenergic neuromodulation in wild-type and presymptomatic SOD1 mice

**DOI:** 10.1016/j.pneurobio.2026.102905

**Published:** 2026-03-10

**Authors:** Stefano Antonucci, Guillaume Caron, Natalie Dikwella, Sruthi Sankari Krishnamurthy, Anthony Harster, Bartosz Wasicki, Hina Zarrin, Aboud Tahanis, Florian olde Heuvel, Simon M. Danner, Albert C. Ludolph, Kamil Grycz, Marcin Bączyk, Daniel Zytnicki, Francesco Roselli

**Affiliations:** a Dept. of Neurology, Ulm University, Ulm, DE, Germany; b Université Paris Cité, CNRS, Saints-Pères Paris Institute for the Neurosciences, Paris, France; c German Center for Neurodegenerative Diseases (DZNE), Ulm, DE, Germany; d Department of Neurobiology and Anatomy, College of Medicine, Drexel University, Philadelphia, United States; e Dept. of Neurobiology, Poznán University of Physical Education, Poland

**Keywords:** β-adrenergic neuromodulation, Homeostatic regulation, G protein-coupled receptors, Channelome, Amyotrophic Lateral Sclerosis, Transcriptomics, *in vivo* intracellular electrophysiology

## Abstract

Homeostatic feedback loops are essential to stabilize the activity of neurons and neuronal networks. It has been hypothesized that, in the context of Amyotrophic Lateral Sclerosis (ALS), an excessive gain in feedback loops might hyper- or hypo-excite motoneurons (MNs) and contribute to the pathogenesis. Here, we investigated how the neuromodulation of MN intrinsic properties is homeostatically controlled in presymptomatic adult SOD1 (G93A) mice and in the age-matched control WT mice. First, we determined that Adrb2 and Adrb3 adrenergic receptors, which are Gs-coupled receptors and subject to tight and robust feedback loops, are specifically expressed in spinal MNs of both SOD1 and WT mice at P45. We then demonstrated that these receptors elicit a so-far overlooked neuromodulation of the electrical properties of MNs, in particular the frequency-current gain, a crucial determinant of excitability. These electrical properties are homeostatically regulated following receptor engagement, which triggers ion channel transcriptional changes and downregulates those receptors. These homeostatic feedbacks are not dysregulated in presymptomatic SOD1 mice, and they set the MN excitability upon β-adrenergic neuromodulation.

## 1. Introduction

Homeostatic plasticity is an essential mechanism that stabilizes neuronal network activity by regulating neuronal excitability (Turrigiano, 1999; Marder and Goaillard, 2006). Intrinsic excitability is set by a large panoply of ion channels (Smith and Brownstone, 2022) that are not only regulated at transcriptional level but are further subject to neuromodulation that controls their biophysical properties through post-translational modification (such as phosphorylation; Reckling *et al.*, (2000). Indeed, neuromodulation acts through G protein-coupled membrane receptors (GPCRs) that activate or inactivate a number of intracellular kinases and G protein subunits targeting ion channels (Carret al., 2003; Luo et al., 2022; Gonzalez-Hernandez et al., 2024). However, signaling of the GPCRs itself is subject to multiple feedback loops. A well-established example is the desensitization of the β-adrenergic receptors, mainly coupled to the Gs protein, during prolonged exposure to their agonists (Maaliki et al., 2024). Their desensitization can be caused by several mechanisms involving surface localization, protein stability and transcriptional downregulation and mRNA stability (Bouvier et al., 1989; Okeke et al., 2019). Upon activation of the receptor, the Gs protein activates the cAMP/PKA pathway, inducing phosphorylation of the GPCR itself and, as a consequence, the recruitment of β-arrestins on the adrenoceptor and the endocytosis of the complex (Maaliki et al., 2024). Prolonged agonist exposure can also induce a decreased transcription or mRNA stability, reducing mRNA abundance (Bouvier et al., 1989). Receptor desensitization results in a reduced neuromodulation, which represents a short-term feedback that contributes to overall homeostatic plasticity.

In Amyotrophic Lateral Sclerosis (ALS), vulnerable spinal motoneurons (MNs) and the neuronal networks that control their firing display abnormalities from early motor presymptomatic phases onwards (Bączyk et al., 2022). Indeed, in the SOD1(G93A) ALS murine model (henceforth, SOD1), alterations of intrinsic electrical properties of MNs and synaptic inputs to MNs are evolving from embryonic to juvenile and adult time points (Bączyk et al., 2020; Branchereau et al., 2019; Cavarsan et al., 2023; Delestree et al., 2014; Jensen et al., 2020; Leroy et al., 2014; Martin et al., 2013; Martínez-Silva et al., 2018; Nascimento et al., 2024; Quinlan et al., 2011). While it has been hypothesized that an excessive gain in homeostatic feedback loops (Brownstone and Lancelin, 2018; Kuo et al., 2020) might hyper- or hypo-excite MNs and drive the pathogenesis of ALS, the occurrence of actual alterations of homeostatic responses of ALS MNs has not been demonstrated.

To test this hypothesis, we investigated if the neuromodulation of MN intrinsic properties is homeostatically controlled in presymptomatic adult SOD1 mice at the degeneration onset of neuromuscular junctions (about P50, Pun et al., 2006), and age-matched wild-type (WT) controls. First, we determined which Gs-coupled receptors are specifically expressed in MNs and are not dysregulated in adult presymptomatic SOD1 mice. Among them, we found Adrb2 and Adrb3 β-adrenergic receptors, encoded respectively by *Adrb2* and *Adrb3* genes, and known to be subject to tight and robust feedback loops. We then studied *in vivo* how these receptors increased MN firing, through cAMP/PKA activation, both in WT and SOD1 MNs, showing a so far overlooked neuromodulation of MNs through β-adrenergic receptors. Most importantly, we demonstrated that, upon agonist-driven chronic activation, both *Adrb2* and *Adrb3* underwent homeostatic downregulation, desensitizing MNs to the β-adrenergic neuromodulation. However, this homeostatic response is the same in MNs from SOD1 and WT mice, indicating that this form of homeostatic plasticity is not dysregulated at the degeneration onset, which therefore is not linked to an altered homeostasis of β-adrenergic neuromodulation

## 2. Results

### 2.1. Expression of β-adrenergic receptors in MNs of WT and ALS presymptomatic mice

**Fig. 1.**
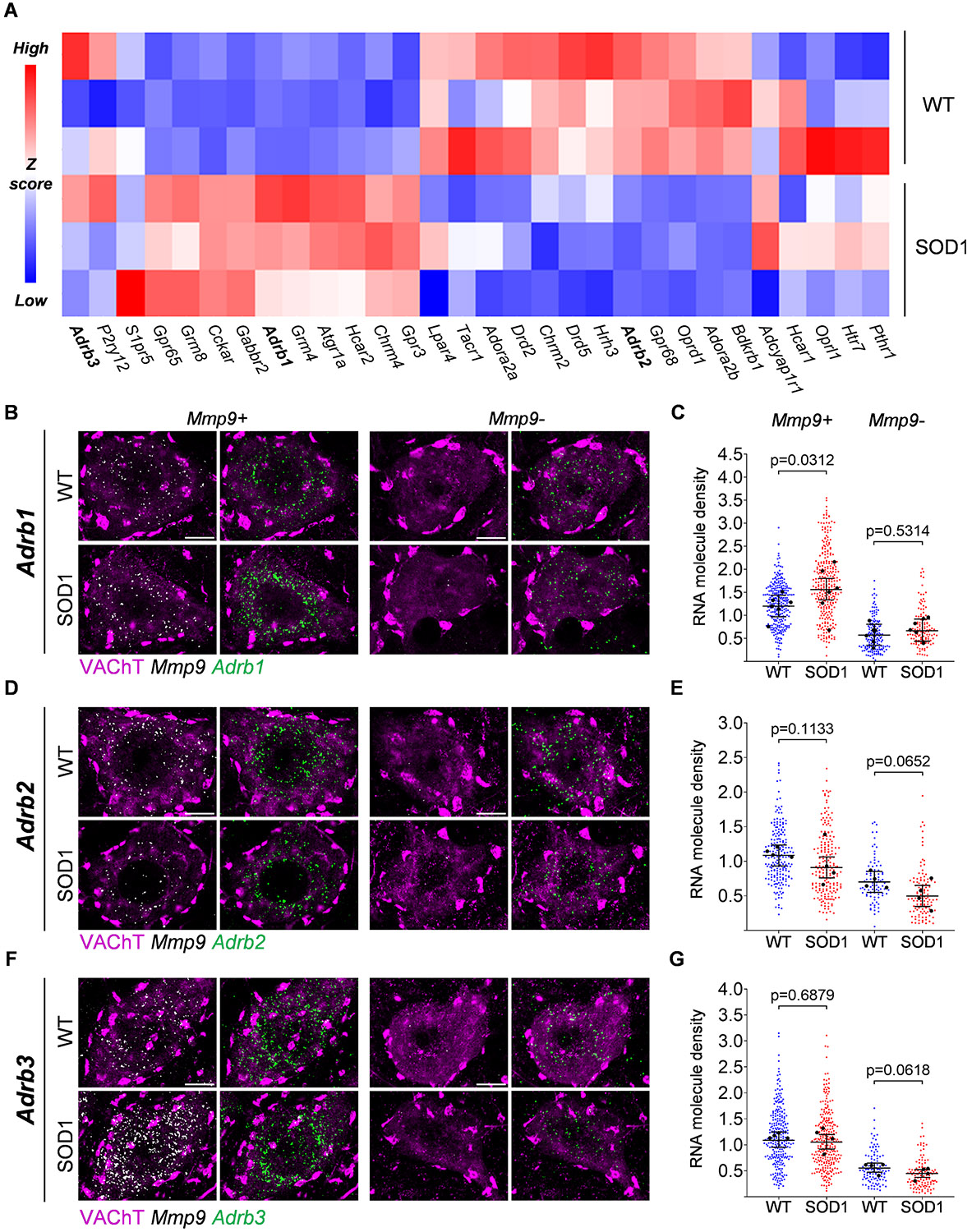
Adrenergic β receptor-encoding *Adrb2* and *Adrb3* genes are expressed in WT and ALS motoneurons and minimally affected at presymptomatic stage. A) GPCR-targeted transcriptomics in laser-microdissected MNs reveals that 9/30 GPCRs are downregulated in SOD1 MNs (*Oprd1*, *Adora2b*, *Bdkrb1*, *Drd5*, *Chrm2*, *Adrb2*, *Gpr68, Drd2, Hrh3),* 10/30 are upregulated (*Gpr65*, *Grm8*, *Cckar*, *Gabbr2*, *Adrb1*, *Grm4*, *Hcar2*, *Atgr1a*, *Gpr3* and *Chrm4*) and 11/30 GPCR (*Adcyap1r1, Hcar1*, *Oprl1*, *Pthr1*, *Htr7*, *Adrb3*, *P2ry12*, *Lpar4*, *S1pr5, Tacr1* and *Adora2a*) are comparably expressed. Of note, *Adrb2* and *Adrb3* are minimally affected (*blue*, downregulated genes; *red*, upregulated genes). 30 MNs were pooled per animal; N = 3 animals per group. B-C) Single-molecule *in situ* hybridization (ISH) confirms the upregulation of *Adrb1* mRNA in *Mmp9*+ (vulnerable), but not in *Mmp9*− (resistant) MNs. D-G) ISH reveals the expression of *Adrb2* (D-E) and *Adrb3* (F-G) in WT and SOD1 MNs. All confocal images show single representative MNs (left panel, merged VAChT and *Mmp9* mRNA; right panel, merged VAChT and adrenoceptor mRNA). Scale bar = 10 μm. Dotplots quantify individual cell expression levels as the number of mRNA molecules per μm^2^. Small dots are individual MN data points and large black dots indicate average expression per animal; bars represent mean and 95% confidence intervals. N = 4–7 mice per group.

Since β-adrenergic receptors, as well as multiple other PKA-coupled GPCRs, might be subject to different degrees of disease-related modulation and disease-associated transcriptional changes, we first set out to verify their pattern of expression at P45 (presymptomatic stage; Martínez-Silva et al., 2018; Bączyk et al., 2020) in lumbar MNs from SOD1 and WT animals isolated by laser-capture-microdissection (LCM; 30 MNs > 500μm^2^ from L4-L5, as in Song et al., 2023) (Fig. 1A). Notably, the genes encoding for β-adrenergic receptors (*Adrb1–3*) were highly expressed in MNs and either upregulated (*Adrb1*) or showing minimal downregulation (*Adrb2*) or unmodified expression (*Adrb3*) in ALS MNs. We then focused on this receptor family because of their well-known desensitization property upon prolonged agonist exposure and the lack of knowledge of their physiological role in MNs.

We validated the expression of *Adrb1–3* by single-molecule *in situ* hybridization (ISH) in lumbar MNs, distinguishing MN subtypes according to *Mmp9* expression (*Mmp9*+ MNs are disease-vulnerable innervating Fast-contracting Fatigable (FF) and large Fast-contracting Fatigue-Resistant (FR) motor units, whereas *Mmp9*− MNs are disease-resistant innervating small FR and Slow-contracting (S) motor units; Kanning et al., 2010; Kaplan et al., 2014; Leroy et al., 2014). All MNs variably express *Adrb1–3* mRNAs. However, *Adrb1–3* mRNA was more abundant in *Mmp9*+ than in *Mmp9−* MNs (50% or fewer mRNA molecules in *Mmp9−* MNs; Fig. 1B-G). The quantification of mRNA molecules density in *Mmp*9 + and *Mmp9*− MNs revealed a pattern of expression of *Adrb1–3* compatible with the transcriptome data: *Adrb1* was upregulated in *Mmp9* + (but not in *Mmp9*−; Fig. 1B-C), *Adrb2* showed no change in *Mmp9* + and a trend toward downregulation in *Mmp9−* (Fig. 1D-E) and *Adrb3* was unaltered in *Mmp9* + and showed a trend toward downregulation in *Mmp9−* MNs (Fig. 1F-G; all statistics are reported in Supplemental Excel Table 1).

Although all three β-adrenergic receptors could be amenable targets to explore their homeostatic desensitization, agonists for Adrb1 receptor either have poor blood-brain-barrier penetration or limited selectivity, and substantial systemic effects (Yi et al., 2017). On the other hand, highly selective and brain-permeant agonists are available for Adrb2 and Adrb3 receptors. Because of the favourable pharmacology and the lack of downregulation at baseline (P45) in *Mmp9* + (vulnerable) MNs, Adrb2 and Adrb3 receptors are then amenable to be probed in the context of their homeostatic loops.

### 2.2. Acute administration of Adrb2 and Adrb3 agonists activates PKA signaling and expression of immediate-early genes

**Fig. 2.**
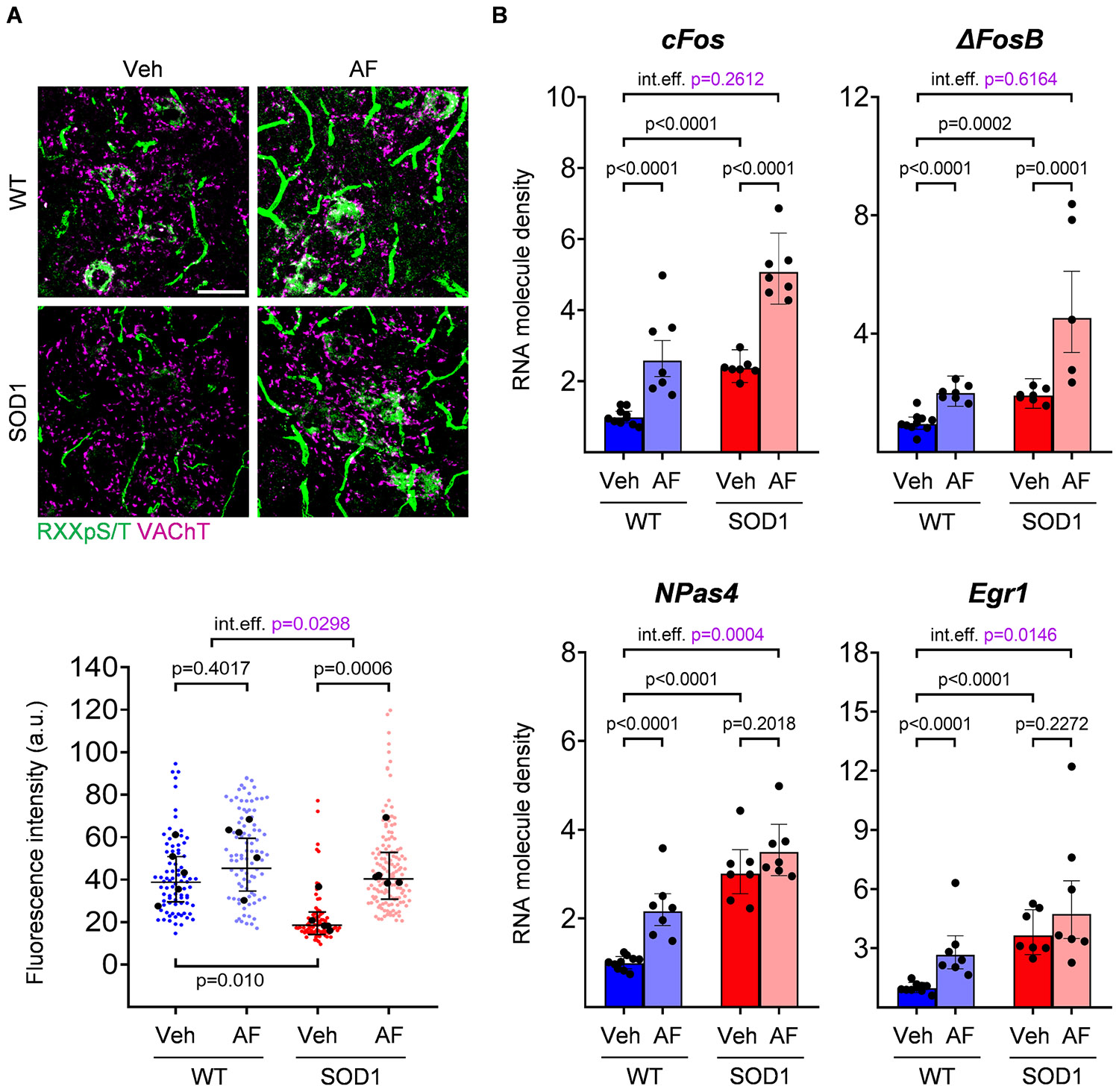
Single-dose pharmacological activation of Adrb2/Adrb3 receptors activates PKA signaling and induces immediate-early gene transcription in motoneurons. A) Immunofluorescence reactivity for the consensus PKA-phosphorylated sequence RRxpS/T reveals increased immunoreactivity in the cytoplasm of MNs and in spinal vasculature upon Adrb2/Adrb3 stimulation in WT (strong trend, significant when the outlier animal is excluded) and SOD1 animals (scale bar = 50 μm). In scatterplots, small dots correspond to individual MN data points, large black dots to average per animal; mean and 95% confidence intervals are depicted. N = 5 animals per group. Significances on top bars illustrate interaction effects (magenta). *Post-hoc* significances are shown for WT Veh *vs.* WT AF (treatment effect in WT), SOD1 Veh *vs.* SOD1 AF (treatment effect in SOD1). B) Induction of immediate-early gene mRNAs (*cFos*, *ΔFosB*, *NPas4*, *Egr1*) in MNs upon Adrb2/Adrb3 stimulation. Each dot represents an individual animal. Significance on top bars illustrate interaction effects (magenta). *Post-hoc* significances are shown for WT Veh *vs.* WT AF (treatment effect in WT, lower left bar), SOD1 Veh *vs.* SOD1 AF (treatment effect in SOD1, lower right bar) and for WT Veh *vs.* SOD1 Veh (genotype effect before treatment, middle bar). N = 7–10 animals per group.

Prior to their use to study β-adrenergic desensitization feedback in MN, we demonstrated that two FDA-approved adrenergic Adrb2 and Adrb3 agonists (formoterol and amibegron, respectively) cross the blood-brain barrier and engage signal transduction in MN, and can elicit a transcriptome modulation in MNs. As detailed above, Adrb1 agonists with suitable selectivity and blood-brain-barrier penetration are lacking. WT or SOD1 mice were administered Adrb2/3 agonists (noted AF in all figures) or vehicle, and PKA cascade activation was assessed at 3 h in spinal cord sections by immunolabeling for the PKA-phosphorylation consensus motif RRxpS/T. In vehicle-treated SOD1 animals (baseline PKA activity), MNs displayed a lower RRxpS/T immunostaining than WT (Fig. 2A), possibly related to the reduced synaptic signaling (Baczyk *et al.*, 2020). However, agonist treatment elicits a substantial increase in RRxpS/T immunoreactivity in SOD1 animals (Fig. 2A), indicating target engagement (Supplemental Excel Table 1). In WT type animals, no significant difference in RRxpS/T was detected, an effect driven by the higher levels of baseline phosphorylation and by one non-responding animal (trend toward increased RRxpS/T is observed upon removal of this outlier; nevertheless this datapoint was not excluded from the analysis; Fig. 2A). Coherent with the signaling engagement, in LCM-dissected MNs, Adrb2/Adrb3 agonists upregulated the expression of multiple immediate-early genes both in WT and SOD1 animals (3 h after treatment; in particular, *cFos*, *ΔFosB*, *Npas4* and *Egr1* were induced in WT and *Fos*, *ΔFosB* in SOD1 animals; Fig. 2B and Supplemental Excel Table 1). Of note, the baseline expression levels of immediate-early genes was higher in SOD1 than in WT, possibly indicating the MN response to pathogenic pathways unrelated to adrenergic signaling (such as oxidative stress and neuroinflammation; Sharp and Sagar, 1994). Taken together, these data demonstrate that Adrb2/Adrb3 receptors on murine MNs are engaged by agonists and linked to signaling and transcriptional responses.

### 2.3. Acute activation of Adrb2/Adrb3 receptors increases MN excitability in both WT and SOD1 mice

The neuromodulatory properties on MNs of the Adrb2 and Adrb3 adrenergic receptors are unknown so far. Then, before investigating the homeostatic responses triggered by a long exposure to agonists, we needed to elucidate how intrinsic electrical properties of MNs are modulated by acute delivery of the agonists. Our physiological studies relied on *in vivo* intracellular recordings carried out on anesthetized WT and SOD1 mice. We randomly recorded MNs from hindlimb muscles innervated by the tibial and common peroneal nerves (Materials and Methods, Section 5.3). To investigate the neuromodulatory properties, we first recorded, in each experiment, a sample of hindlimb MNs (between 1 and 7), then we intravenously (i.v.) delivered a single bolus of the Adrb2/Adrb3 agonists (AF) before recording a new sample of MNs (between 1 and 9) in the next 3 h following the drug delivery (see Methods section).

**Fig. 3.**
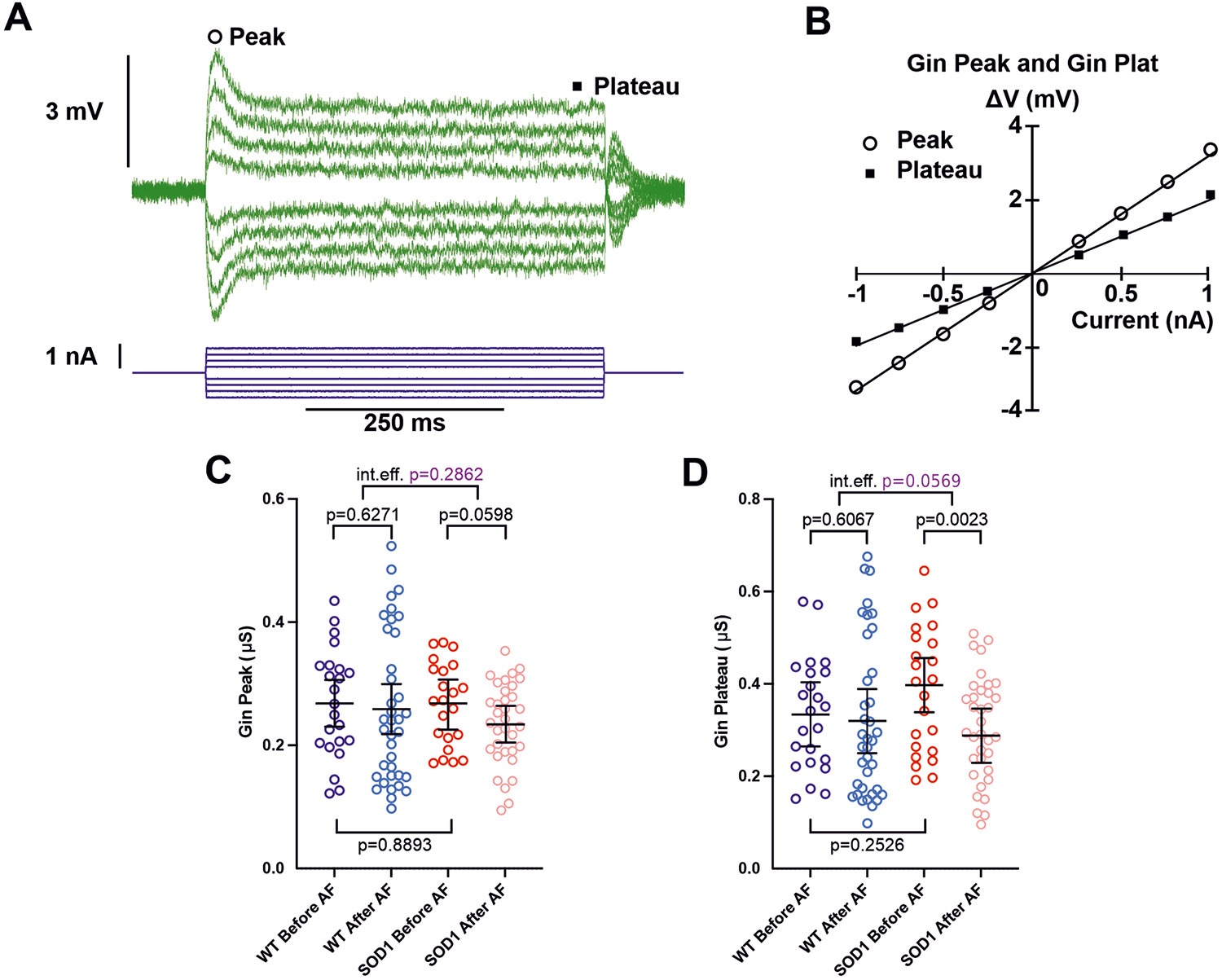
Single-dose delivery of Adrb2/Adrb3 agonists decreases the plateau input conductance of motoneurons in SOD1 mice. A) Average responses of a SOD1 MN to a series of current pulses lasting 500 ms and ranging from −1 to +1 nA. Notice that, in each voltage response, the peak is followed by a sag before it stabilizes at a plateau. B) Plot of the voltage-current response measured at peak and plateau against the intensity of current. Effect of Adrb2/Adrb3 agonists on peak input conductance (C) and plateau input conductance (D) in MNs from WT and SOD1 mice. Significances on top bars illustrate the lack of interaction effects (magenta). *Post-hoc* significances are shown for WT before AF *vs.* WT after AF (treatment effect in WT), SOD1 before AF *vs.* SOD1 after AF (treatment effect in SOD1) and for WT before AF *vs.* SOD1 before AF (genotype effect before treatment, bottom bar). Amibegron + Formoterol (AF). N = 6 WT mice and N = 11 SOD1 mice.

**Fig. 4.**
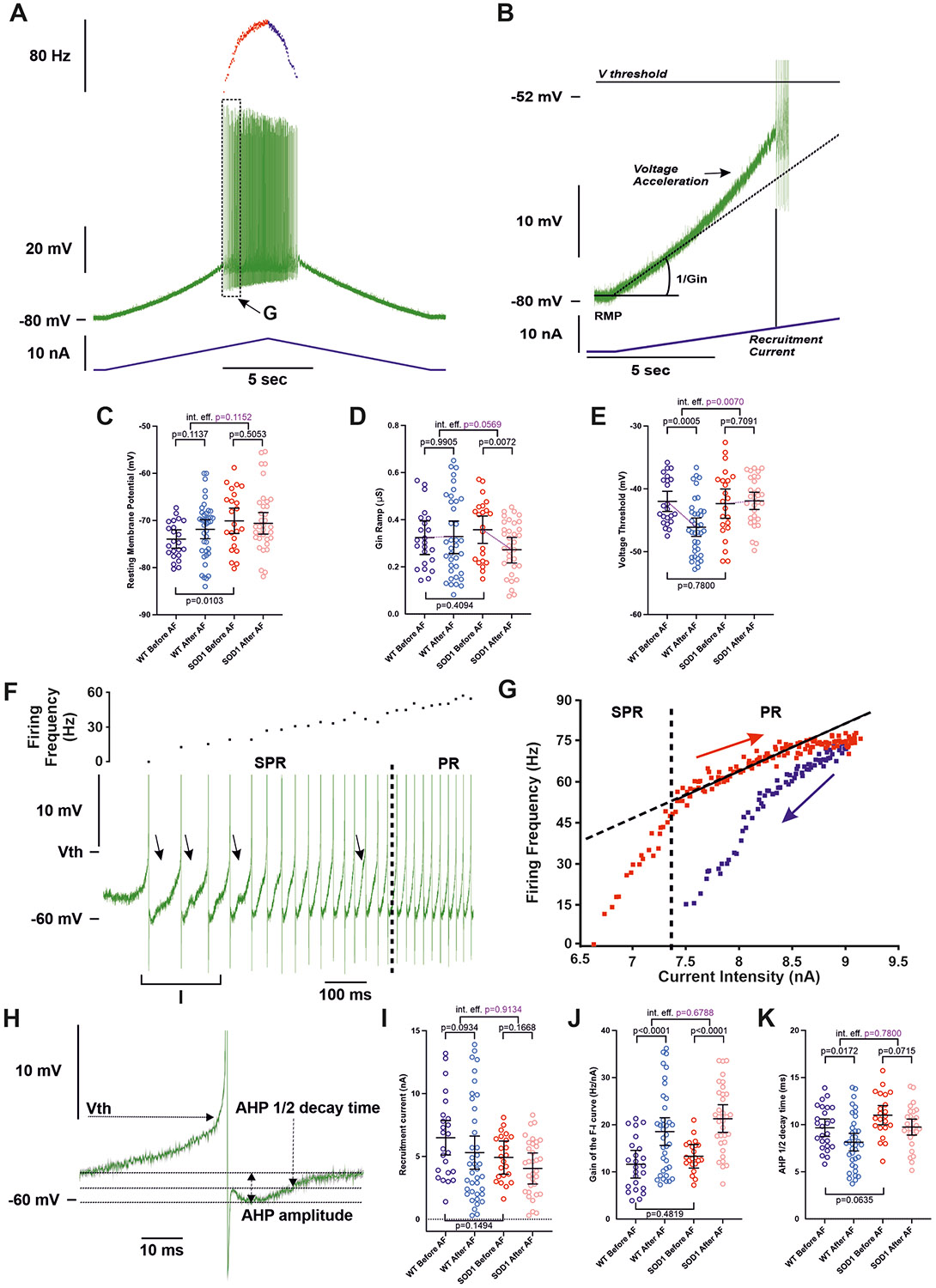
Acute delivery of Adrb2/Adrb3 agonists increases the firing of motoneurons in WT and SOD1 mice. A) Representative response of a SOD1 MN to a slow ramp (1 nA/s) of current. Ramp of current (blue bottom trace), voltage response (green middle trace) and instantaneous firing frequency (top trace with ascending leg in red and descending leg in blue). B) Magnification of the ramp subthreshold voltage (green top trace) and current (blue bottom trace). Notice that Vthreshold (Vth) was measured in expanded traces as shown in F and H. Effect of an acute delivery of Amibegron/Formoterol (AF) on resting membrane potential (C), ramp input conductance (D), voltage threshold for spiking (E) in MNs from WT and SOD1 mice. F) Magnification and time expansion of the voltage trace from the region indicated in A. At the firing onset, oscillations (arrowheads) appear in the interspike intervals, which characterizes the subprimary range (SPR). When the injected current increases, oscillations disappear and the firing frequency increases linearly (primary range, PR). G) Plot of the instantaneous firing frequency against the intensity of the current for the ascending (red) and descending (blue) legs of the ramp shown in A. The F-I relationship displayed a clockwise hysteresis. Vertical dashed line indicates the transition between the SPR and the PR on the ascending leg. The gain of the F–I curve can be estimated by the slope of the linear regression (continuous line) in the PR. H) Spike-triggered averaging of the three first action potentials displayed in F. In the example shown, the 3 first interspike intervals were 83, 58 and 59 ms, respectively. The AHP half-decay time is the time between AHP peak amplitude and where the AHP relaxed to half its amplitude. On the spike triggered averaging of the first 3 spikes (Fig. 4H), an AHP is clearly visible with a half-decay of 9.6 ms, *i.e.* at least 6 times shorter than the interspike intervals allowing the measurement of AHP half decay-time before the next spike. Effect of an acute delivery of Amibegron/Formoterol (AF) on the recruitment current (I), Gain of the F-I relationship (J), AHP half-decay time (K) in MNs from WT and SOD1 mice. In all graphs, each point represents one MN and the mean ± 95% confidence intervals are shown. Significances on top bars illustrate interaction effects (magenta). *Post-hoc* significances are shown for WT before AF *vs.* WT after AF (treatment effect in WT), SOD1 before AF *vs.* SOD1 after AF (treatment effect in SOD1) and for WT before AF *vs.* SOD1 before AF (genotype effect before treatment, bottom bar). Amibegron + Formoterol (AF). N = 6 WT mice and N = 11 SOD1 mice.

A series of depolarizing or hyperpolarizing pulses of currents (Fig. 3A-B) and a triangular slow ramp of current (Fig. 4A-B) allowed us to investigate whether the treatment with Adrb2/Adrb3 agonists modify the physiological parameters that determine MN excitability (resting membrane potential, input conductance, voltage threshold for firing, recruitment current, firing gain; all statistics are detailed on Supplemental Excel Table 1). We found that the treatment does not significantly change the resting membrane potential both in WT and SOD1 MNs (Fig. 4C), nor the input conductance measured on the response peak (Gin Peak) at the onset of current pulses (Fig. 3C). In response to long pulses, the peak is followed by a sag down to a plateau that is caused by an H-current (Fig. 3A) (Ito and Oshima, 1965; Takahashi, 1990; McLarnon, 1995; Manuel et al., 2009). We then also measured the input conductance at the end of the plateau (Gin plateau, Fig. 3A-B) and we found that the treatment significantly reduces the Gin plateau in SOD1 but not in WT mice (Fig. 3D, the interaction effect is close to the significance threshold; p = 0.057). In the same line, the input conductance measured at the beginning of the slow ramp (Gin ramp, Fig. 4B) is reduced by the treatment in SOD1 but not WT mice (interaction effect; p = 0.045, Fig. 4D). It then appears that β agonists decrease Gin plateau and Gin ramp in MNs from SOD1 mice, likely through a modulation of H-current. This effect might increase MN excitability during slow depolarizing events.

Another parameter that sets MN excitability is the voltage threshold at which spikes are fired (Vthreshold): the more hyperpolarized the voltage threshold for spiking, higher is the probability to elicit firing. We found that treatment causes a significant hyperpolarization of the voltage threshold in WT mice, suggesting an increased excitability, but not in SOD1 mice (Fig. 4E). The next important parameter for assessing excitability is the amount of depolarizing current required to reach the firing threshold, *i.e.* the recruitment current. During slow ramps, the voltage trajectory to reach the firing threshold is not linear since an acceleration occurred below the threshold for firing (Fig. 4B, arrow). We found that treatment has no significant effect on the recruitment current both in WT and SOD1 mice (Fig. 4I).

Once the discharge is initiated, the MN starts to discharge in the mixed mode oscillations regime, in which small oscillations (arrows in Fig. 4F) are present between spikes, describing a subprimary range, before transitioning to a regime of firing where spike frequency increases linearly with the injected current, *i.e.* the primary range (Fig. 4G, and see Manuel et al., 2009). Another important criteria to assess MN excitability is the slope (frequency-current gain) of the primary firing range (Fig. 4G), with a higher frequency-current intensity gain (F-I gain) indicating that the MN is more excitable. Indeed, the strongest action of Adrb2/Adrb3 agonists was observed on the F-I gain that is very significantly increased in MNs from both WT (64% increase) and SOD1 mice (63% increase) (Fig. 4J).

It was established, both theoretically and experimentally, that the F-I gain in the primary firing range is proportional to the reciprocal of the AHP relaxation time constant (not of the AHP total duration): shorter the time constant, higher the gain (Kernell, 1999; Meunier and Borejsza, 2005; Manuel et al., 2006). In contrast, the firing frequency in the sub-primary firing range is not controlled by the AHP because the AHP has fully, or almost fully, relaxed before the next spike (Manuel et al., 2009, Iglesias et al., 2011). This feature makes it possible to measure the AHP half-decay time in the sub-primary range to estimate the possible changes in AHP kinetics. Of note, the AHP half-decay time is easier to measure than the AHP relaxation time constant but they are, on average, close to each other (see Table 2 in Manuel et al., 2009). We averaged the AHP following the 3–5 spikes with the longest interspike intervals in the sub-primary range and we measured the AHP half-decay time on the averaged trace (see Materials and Methods, Section 5.3; Fig. 4H). Interestingly, the treatment with Adrb2/Adrb3 agonists caused a significant shortening of the AHP half-decay time in WT MNs, and an almost significant shortening (p = 0.07) in SOD1 MNs (Fig. 4K). The treatment has then accelerated the AHP kinetics in the sub-primary range. It is likely that a similar acceleration of AHP kinetics took place also in the primary range, which may contribute to the observed increase in the F-I gain of the primary range.

To sum up, an acute administration of Adrb2/Adrb3 agonists modulates MN excitability mainly through an increase of the F-I gain in both WT and SOD1 mice. However, a decrease of Gin plateau or Gin ramp in MNs from SOD1 mice, and a hyperpolarization of voltage threshold for spiking in MNs from WT mice may also contribute to the excitability increase induced by Adrb2/Adrb3 agonists.

### 2.4. The cAMP/PKA pathway contributes to the Adrb2/Adrb3 neuromodulation of MN excitability in SOD1 mice

To confirm that the increase in the F-I gain of MNs upon acute activation of the Adrb2/Adrb3 receptors was mediated by the cAMP/PKA pathway (and not to alternative signaling, such as β-arrestin/ERK; Huang et al., 2018), we compared the impact of Adrb2/Adrb3 agonists on the F-I gain in the absence or in the presence of H89 inhibitor of PKA (see Materials and Methods, Section 5.6). In the absence of H89, we confirmed again that Adrb2/Adrb3 agonists increase the firing gain (54% increase), but it was not increased anymore in the presence of H89 (Supplemental Fig. 1), suggesting that PKA is involved in the β-adrenergic neuromodulation of the firing gain.

**Fig. 5.**
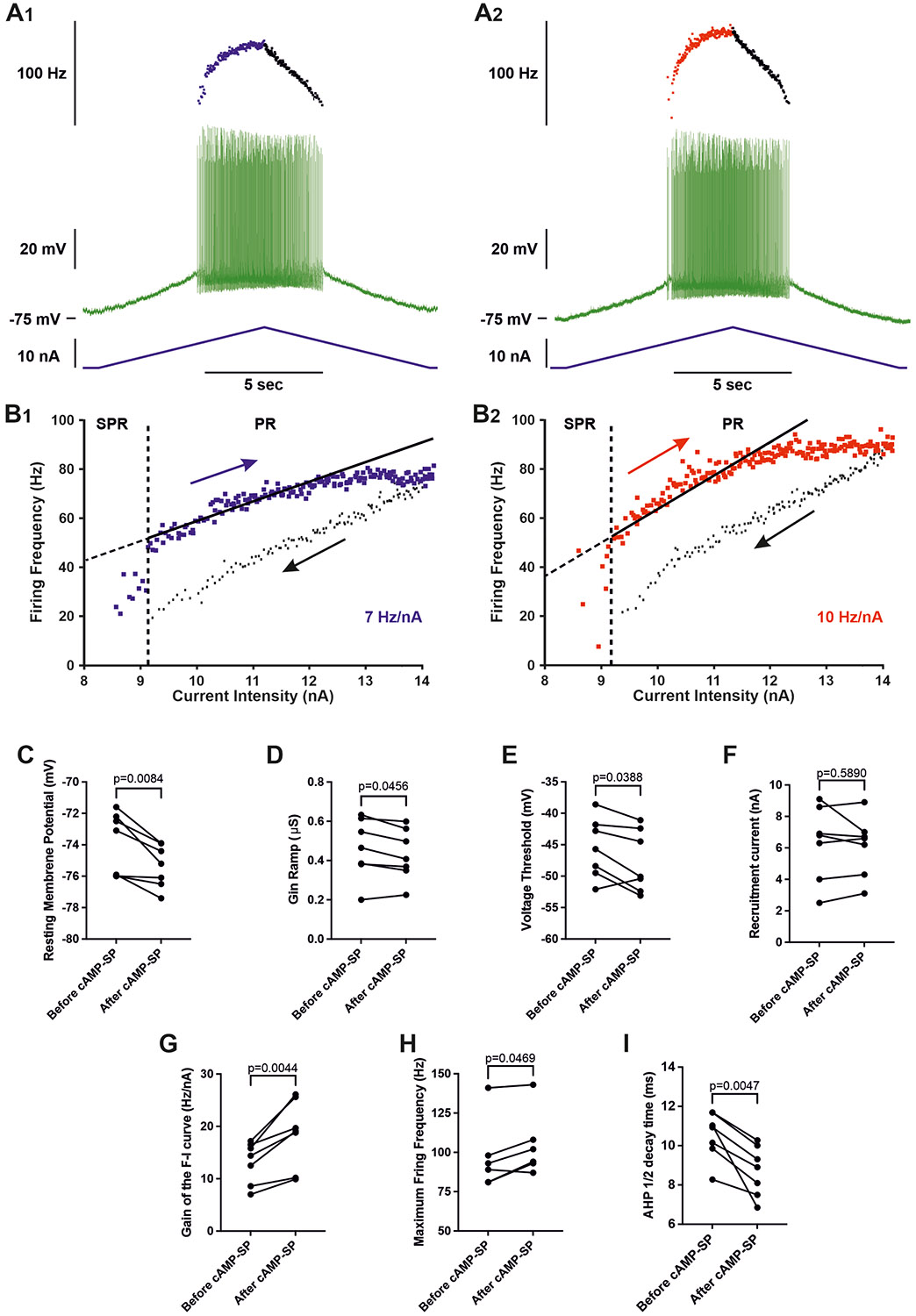
Single-dose and direct activation of cAMP/PKA pathway increases the firing of motoneurons in SOD1 mice. A) Representative response of a SOD1 MN to a slow ramp (2 nA/s) of current, before (A_1_) and 12 min after (A_2_) the iontophoretic injection of cAMP-SP in the same MNs. The ramp of current (blue bottom trace), voltage response (green middle trace) and instantaneous firing frequency (top trace with ascending leg in blue for A_1_ or red for A_2_, and descending leg in black for both A_1_ and A_2_. B) Plot of the instantaneous firing frequency against the intensity of current for the ascending (blue for B_1_ or red for B_2_) and descending legs (black for both B_1_ and B_2_) of the ramps shown in A. The F-I relationships displayed a clockwise hysteresis. Vertical dashed line indicates the transition between the SPR and the PR on the ascending branch. The gain of the F–I relationship can be estimated by the slope of the linear regression (continuous line) in the PR. C-I) Comparison of electrophysiological properties extracted from the slow ramp of current before and after injection of cAMP-SP. Before values are measured just before the injection; After values are averages of the recordings repeated after the iontophoretic injection of cAMP-SP; n = 7 MNs recorded in N = 7 SOD1 mice. C) Resting membrane potential. D) Ramp input conductance. E) Voltage threshold for spiking. F) Recruitment current. G) Gain of the F-I curve. H) Maximum firing frequency reached at the end of the ascending ramp (the velocity and amplitude were the same for all ramps in each MN). I) AHP half-decay time. In all graphs, each linked two points represent one MN. Before *vs*. after values were compared using a paired *t*-test except for maximum firing frequency, for which a Wilcoxon paired test was used.

However, H89 may also inhibit other kinases besides PKA (Lochner and Moolman, 2006). Therefore, to prove the major role of PKA in this type of neuromodulation, we increased the PKA activity directly by intracellular injection of the cAMP analogue in MNs (cAMP-SP; Bączyk et al., 2020). Since the cAMP analogue is delivered intracellularly in the MNs, this experiment also allowed us to rule out off-target effects (such as increase in heart rate, etc…). We recorded the responses of individual MNs to a slow ramp of current and to long pulses (in order to determine the peak and the plateau input conductances) before and for at least 10 min after we electrophoretically injected cAMP-SP through the intracellular microelectrode (normalized electrical load 2600 ± 600 nA. sec/μS). The whole procedure required that the MN recording did not deteriorate for at least 30 min, a condition that limited the number of successful experiments (see Materials and Methods, Section 5.4). We have been able to follow the effect of cAMP-SP in 7 MNs from SOD1 mice (Fig. 5, statistics in Supplemental Excel Table 1). We found that cAMP-SP significantly increased the F-I gain (average increase 32%, Fig. 5 G) and, consequently, the maximal firing frequency measured at the end of the ramps (ramp velocity and amplitude were kept constant before and after cAMP-SP injection for each individual motoneuron) (Fig. 5H). In addition, cAMP-SP shortened the AHP half-decay time as Adrb2/Adrb3 agonists did (Fig. 5I). cAMP-SP also induced a small hyperpolarization of the resting membrane potential (Fig. 5 C) and of the voltage threshold for spiking (Fig. 5E), and a slight decrease of the input conductance measured on the ramp (Gin Ramp, Fig. 5D). Altogether these two series of experiments strongly suggest that the cAMP/PKA pathway contributes to the neuromodulatory effects in MNs mediated by the Adrb2/Adrb3 agonists.

### 2.5. Acute delivery of Adrb2 and Adrb3 agonists is sufficient to induce a dysregulation of ion channels transcription

**Fig. 6.**
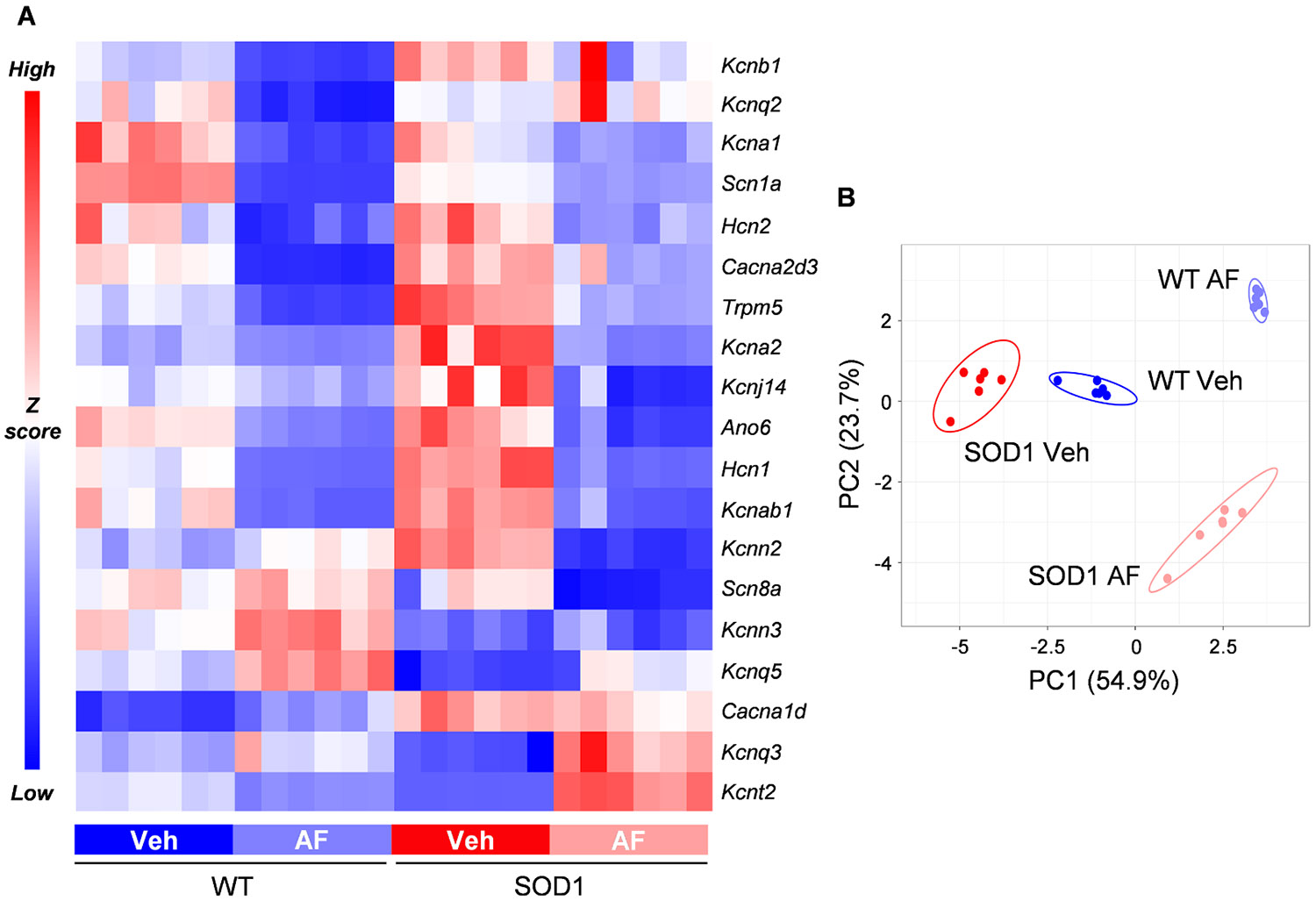
Single-dose pharmacological activation of Adrb2/Adrb3 receptors modulates ion channel transcription in motoneurons. A) Targeted ion-channel transcriptome in WT and SOD1 MNs reveals the broad downregulation of multiple channels upon Adrb2/Adrb3 stimulation (downregulation*: blue*; upregulation: *red*). N = 6 mice per experimental group. B) PCA plot of ion channel gene transcription reveals the separation of experimental groups according to drug treatment and genotype. N = 6 mice per experimental group.

Since Adrb2/Adrb3 agonists exerted a transcriptional modulation already with 3 h of administration (as shown by the induction of immediate-early genes), we explored whether they would also influence the genes encoding for ion channels responsible for MN excitability. We investigated the expression of 19 ion channel subunit-encoding genes that are reported to be enriched in MNs (according to the Allen Spinal Cord gene expression Atlas; Henry and Hohmann, 2012) and that are responsible for K^+^ (*Kcna1, Kcna2, Kcnab1, Kcnb1, Kcnj14*, *Kcnn2*, *Kcnn3*, *Kcnq2*, *Kcnq3*, *Kcnq5*, *Kcnt2*), Na^+^ (*Scn1a*, *Scn8a*), Ca^2+^ (*Cacna1d*, *Cacna2d3*), Cl^−^ (*Ano6*) or non-selective cationic currents (*Hcn1, Hcn2, Trpm5*). A large fraction of the channels under analysis exhibited significant disease-driven changes already at this presymptomatic stage (14/19, with only *Cacna2d3*, *Hcn2*, *Kcnq2*, *Scn8a* and *Ano6* remaining unaffected, see Supplemental Excel Table 1). Remarkably, three hours after Adrb2/Adrb3 agonist administration, the majority of investigated channels was further significantly modulated (down- or up-regulated) not only in WT but also in SOD1 MNs (Fig. 6 A), with a clear distinction of genotypes and treatment groups upon principal component analysis (Fig. 6B). Specifically, upon treatment both WT and SOD1 MNs displayed a shared pattern of downregulation of Ca^2+^ (*Cacna2d3*), Na^+^ (*Scn1a*), K^+^ (*Kcna1, Kcna2, Kcnab1*), mixed cationic (*Hcn1, Hcn2, Trpm5*) or Cl^−^ (*Ano6*) channels and upregulation of other K^+^ channels (*Kcnq3, Kcnq5*). Overall, 11 out of 19 investigated genes showed similar modulation of their transcriptional responses in AF-treated WT and SOD1 when compared to vehicle-treated littermates (for detailed statistics, see Supplemental Excel Table 1). *Cacna1d*, *Kcnb1*, *Kcnj14*, *Kcnn3*, *Kcnq2* and *Scn8a* were not significantly affected by the adrenergic agonist treatment in both genotypes whereas only two, *Kcnn2* and *Kcnt2,* showed significant changes in opposite directions for WT and SOD1 genotypes. These data demonstrate that an acute stimulation of Adrb2/Adrb3 receptors is sufficient to elicit a rapid modulation of the MN channelome that is similar in WT and SOD1 animals.

### 2.6. A prolonged delivery of Adrb2/Adrb3 agonists triggers homeostatic feedback loops in MNs from WT and SOD1 presymptomatic mice

**Fig. 7.**
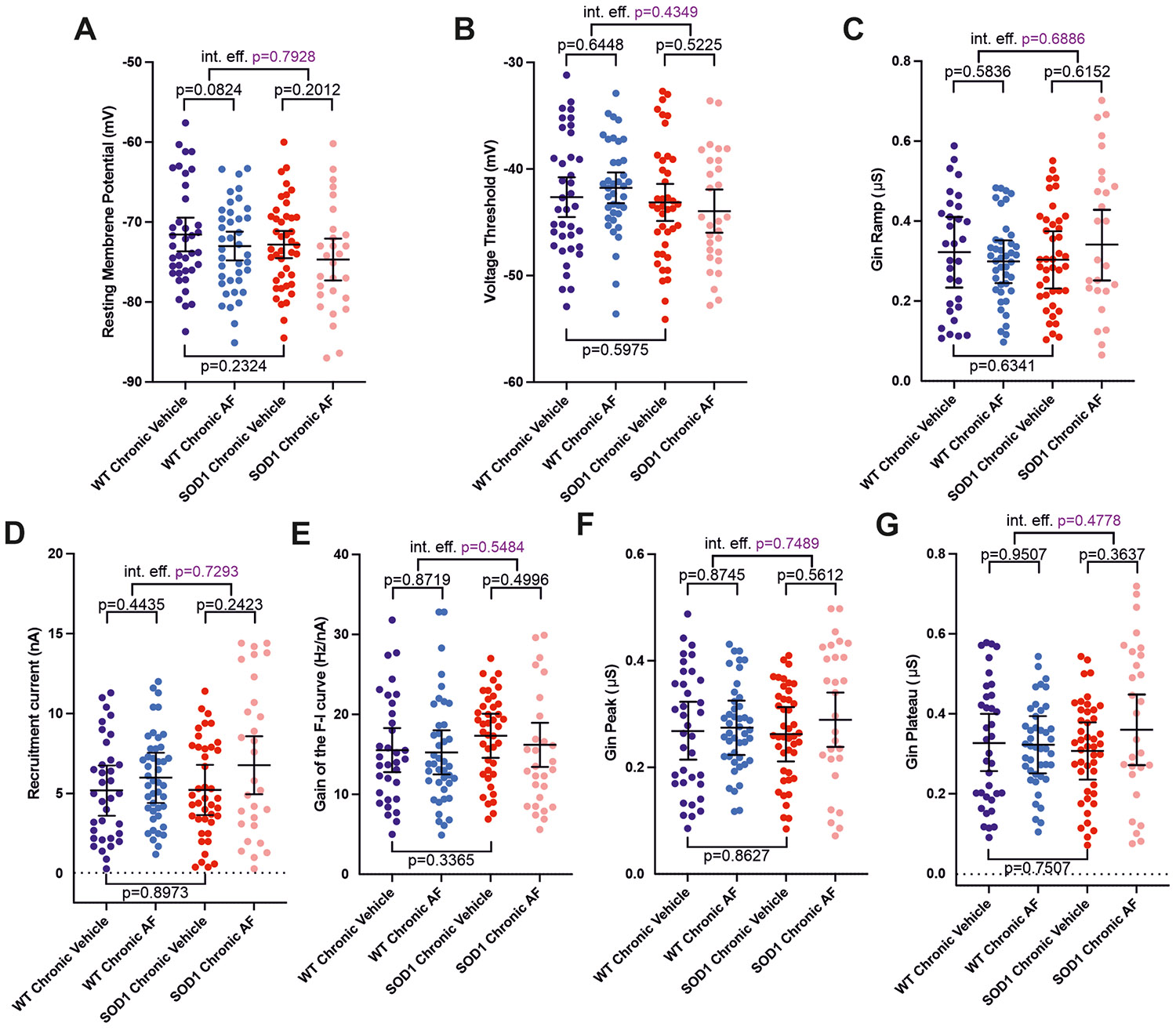
Prolonged delivery of Adrb2/Adrb3 agonists does not increase the firing of motoneurons. A-F) Electrophysiological properties were obtained from slow ramps of current, as in Fig. 4. Effect of the treatment on resting membrane potential (A), voltage threshold for spiking (B), ramp input conductance (C), recruitment current (D), gain of the F-I relationship (E), in MNs from WT and SOD1 mice. F-G) Electrophysiological properties were obtained from a series of current pulses lasting 500 ms, as in Fig. 3. Effect of the chronic treatment on peak input conductance (F), plateau input conductance (G) in MNs from WT and SOD1 mice. In all graphs, each point represents one MN and the mean ± 95% confidence intervals are shown. Significances on top bars illustrate interaction effects (magenta). *Post-hoc* significances are shown for WT Chronic Vehicle *vs.* WT Chronic AF (treatment effect in WT), SOD1 Chronic Vehicle *vs.* SOD1 Chronic AF (treatment effect in SOD1) and for WT Chronic Vehicle *vs.* SOD1 Chronic Vehicle (genotype effect before treatment). N = 11 WT mice and N = 9 SOD1 mice.

After having established how acute activation of the Adrb2 and Adrb3 adrenergic receptors modulate the MN properties, we then performed electrophysiological experiments to investigate whether a prolonged treatment during 10 days elicits homeostatic response and whether this response is similar or not in MNs from WT and SOD1 mice. We found that most of the effects of Adrb2/Adrb3 agonists disappeared upon chronic administration (Fig. 7, all statistics in Supplemental Excel Table 1). RMP (Fig. 7 A), recruitment current (Fig. 7D), Gin peak (Fig. 7 F), Gin plateau (Fig. 7 G), and Gin ramp (Fig. 7 C) were not affected by the chronic drug delivery when compared with the vehicle-treated group. V-threshold was not affected either (Fig. 7B). Most surprisingly, even the F-I gain, that was the most affected in acute conditions, did not change upon chronic conditions (Fig. 7E). To ensure the reproducibility of these findings, we repeated the *in vivo* intracellular recordings in a second independent cohort in a different laboratory while following the same protocol of i.p./i.v. injection, mouse strain, anesthesia, and intracellular recording window. The main findings could indeed be replicated: a single delivery of Adrb2/Adrb3 agonists increased the F-I gain but this effect was lost after a ten days-long administration (Supplemental Fig. 2C and 2F). Altogether, these findings point to a similar homeostatic regulation of the β-adrenergic neuromodulation both in MNs from WT and SOD1 mice.

**Fig. 8.**
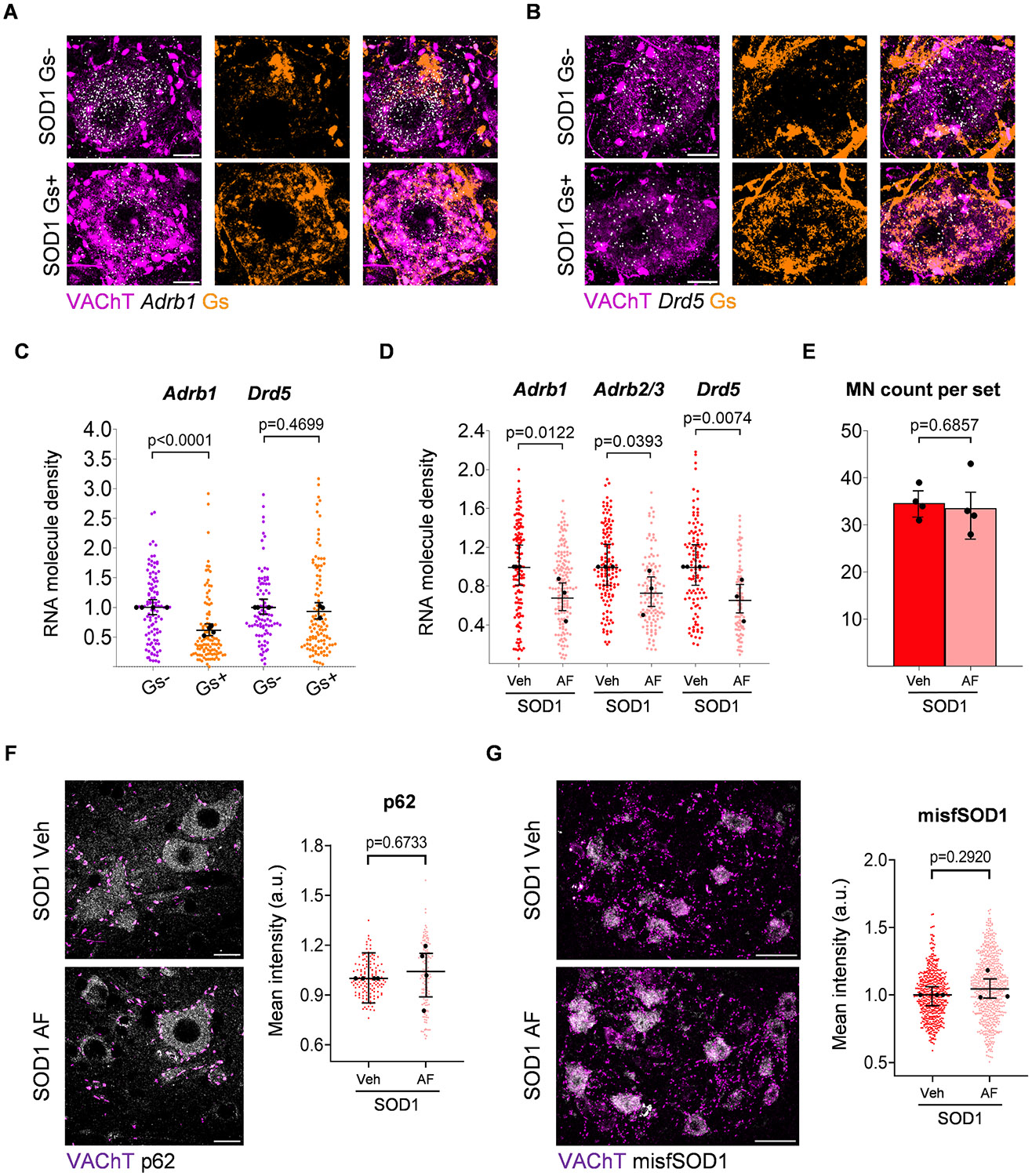
Chronic PKA engagement downregulates PKA-coupled receptors in motoneurons and does not result in amelioration of disease burden. A-C) DREADD(Gs)-mediated chronic chemogenetic activation of PKA pathway in presymptomatic SOD1 MNs results in the downregulation of *Adrb1* and *Drd5* mRNA. Confocal images showcase sample AAV-infected, DREADD(Gs)-expressing (Gs+) or uninfected (Gs-) MNs (from left to right: combined VAChT and receptor mRNA, DREADD(Gs), merge). Scale bar = 10 μm. Individual cell expression levels are quantified as the number of mRNA molecules per μm^2^; N = 4 mice per group for *Adrb1* ISH and N = 3 mice per group for *Drd5* ISH. D) Expression of *Adrb1*, *Adrb2/3* and *Drd5*, revealed by single-molecule *in situ* hybridization, is downregulated by 10 days of Adrb2/Adrb3 agonists administration. mRNA levels are assessed as in A); N = 3 animals per experimental group. E) Ventrolateral MN counts from a set of 3 lumbar spinal cord sections do not change significantly upon chronic Adrb2/Adrb3 agonist administration to p45 SOD1 mice; N = 4 mice per group F) p62 burden in MNs is not altered by 10 days Adrb2/Adrb3 agonist administration. Left panel, representative confocal images of p62 immunostaining in vehicle- or AF-chronically treated lumbar MNs (scale bar = 20 μm). Right panel, quantification of p62 accumulation, assessed as mean cytoplasmic intensity per cell (arbitrary units, a.u.); N = 4 animals per experimental group. G) Misfolded SOD1 burden in MNs is not modified by 10 days Adrb2/Adrb3 agonist administration. Left panel, representative confocal images of misfolded SOD1 immunostaining in vehicle- or AF-chronically treated lumbar MNs (scale bar = 50 μm). Right panel, quantification of misfolded SOD1 accumulation, assessed as mean cytoplasmic intensity per cell (arbitrary units, a.u.); N = 3 mice per group. In all dotplots, small dots correspond to individual MN data points, whereas large dots represent individual animal means. 95% confidence intervals are indicated; the interquartile range is shown in panel E.

In order to elucidate whether a downregulation of the GPCR themselves may contribute to this homeostatic response, we explored the effect on receptor expression either of chronic cAMP/PKA activation or of chronic Adrb2/Adrb3 stimulation. First, we used DREADD-Gs to stimulate cAMP/PKA in MNs (even though it may not fully simulate signaling from endogenous adrenergic receptors; Fig. 8A-C); DREADD expression was obtained by intraspinal injection of AAV9 in SOD1/ChAT-Cre double-transgenic mice (to restrict the expression to MNs; Bączyk et al., 2020). We quantified the expression of *Adrb1* and *Drd5* in DREADD-expressing and DREADD-non-expressing MNs from the same tissue section, so that any non-chemogenetic effect of CNO (such as antagonistic effect on dopaminergic receptors due to back-conversion to Clozapine) would be balanced between the two groups. Upon administration of the cognate agonist (Clozapine-N-Oxide, 10 days), we observed a substantial downregulation of *Adrb1* in MNs (by ISH; Fig. 8A, C) but not of the PKA-coupled *Drd5* (Fig. 8B-C). On the other hand, 10 days-long administration of Adrb2/Adrb3 agonists resulted in the downregulation of *Adrb1*, *Adrb2* and *Adrb3* as well as a trend toward downregulation of *Drd5* genes coding for these receptors (Fig. 8D and Supplemental Excel Table 1). This reduced transcription might be the consequence of receptor desensitization and play a role in preventing the drug from increasing MN excitability. Finally, the number of surviving MNs in the ventrolateral area of the lumbar spinal cord was comparable in animals treated with Adrb2/Adrb3 agonist or vehicle (Fig. 8E and Supplemental Excel Table 1) and chronic Adrb2/Adrb3 agonist treatment did not result in a decrease of misfolded SOD1 burden nor in the accumulation of the autophagy marker p62 (Fig. 8F-G), indicating an absence of therapeutic effect on pathogenic processes.

## 3. Discussion

This study reveals a previously overlooked β-adrenergic neuromodulation of MNs and details mechanisms of homeostatic feedback. Adrb2 and Adrb3 adrenergic receptors modulate MN electrical properties, in particular the FI gain which is a crucial determinant of excitability and which was found to be increased both in WT and SOD1 MNs. However, Adrb2 and Adrb3 adrenergic receptors are homeostatically regulated via transcriptional changes. These changes affect ion channel expression and lead to the downregulation of PKA-coupled receptors, including Adrb2 and Adrb3 adrenergic receptors. Notably, these homeostatic mechanisms function similarly in WT and presymptomatic adult SOD1 mice, indicating that they are not dysregulated at this disease stage.

### 3.1. An overlooked β-adrenergic modulation of spinal MN excitability

Noradrenergic (NA) descending fibers originating from the locus coeruleus and subcoeruleus are long known to innervate the spinal cord, where they were shown to play a role in initiating the locomotion (Jordan et al., 2008). Noradrenaline differentially modulates the activity of spinal interneurons involved in locomotion (Bras et al., 1990; Hammar et al., 2004). Descending noradrenergic fibers also directly contact spinal MNs. Each spinal MN was found to receive more than one thousand NA contacts widely distributed throughout the dendritic arborization (Montague et al., 2013; Maratta et al., 2015). While noradrenergic neuromodulation of MN excitability through α-adrenergic receptors has been documented, neuromodulation through β-adrenergic receptors has so far received little attention. Actually, it was initially reported that MNs are endowed with α-adrenergic receptors but are deprived of β-adrenergic receptors (Nicholas et al., 1996). Many works reported multiple effects of noradrenaline on MN excitability (*e. g.*, membrane depolarization, increased input resistance, hyperpolarization of the voltage threshold for spiking, increase of the F-I gain). These effects, may differ from one motor pool to another depending on its location in the brainstem or the spinal cord, but they were most of the time ascribed to α1 adrenergic receptors, sometime to α2 receptors, but never to β-receptors (see Rekling et al., 2000, for a review). However a more recent investigation demonstrated the presence of Adrb1 adrenergic receptors in lumbar MNs of neonate rats and showed that their pharmacological activation decreased the F-I gain (Tartas et al., 2010). To our knowledge, the present study is the only one that investigated the modulation of MN excitability by Adrb2/Adrb3 receptors. We demonstrated that FDA-approved Adrb2/Adrb3 receptor agonists increase the excitability of MNs both in WT and in presymptomatic SOD1 adult mice, primarily through an increase of the F-I gain and, secondary, through a decrease of the stationary input conductance (Gin ramp, Gin plateau) in SOD1 mice and a hyperpolarization of the voltage threshold for spiking in WT mice.

### 3.2. Neuromodulation of mAHP through Adrb2/Adrb3 receptors

It was established that the medium afterhyperpolarization (mAHP), which follows each action potential, is a potent controller of the MN firing, in particular of the gain of the F-I relationship in primary range (Kernell, 1999; Manuel et al., 2006). Indeed, in simple MN models, the F-I gain in primary range depends on the inverse of the product between AHP conductance, AHP relaxation time constant and AHP driving force (Meunier and Borejsza, 2005; Manuel et al., 2006). The smaller and faster the mAHP conductance, the higher is the F-I gain in the primary range. We found here that Adrb2/Adrb3 receptors agonists shortened the AHP half-decay time (a parameter close to the AHP relaxation time constant (Manuel et al., 2009)) in the sub-primary firing range. It is then very likely that the Adrb2/Adrb3 agonists also accelerated the AHP kinetics in the primary range, which may, at least partially, contribute to the F-I gain increase in this firing range. Furthermore, Adrb2/Adrb3 agonists reduce the voltage threshold for spiking in MNs from WT mice decreasing the AHP driving force that contributes to increase the F-I gain in these MNs. A secondary determinant of the frequency-current gain is the plateau input conductance (Meunier and Borejsza, 2005) that was found to be reduced by Adrb2/Adrb3 agonists in MNs from SOD1 mice. In total the reduced AHP half-decay time, voltage threshold for spiking and Gin plateau may all contribute to the increased frequency-current gain.

The mAHP current in rodent MNs is mainly caused by the SK2 and SK3 calcium-dependent potassium channels, which were found to cluster on soma and proximal dendrites of α-MNs at the level of C-boutons (Deardorff et al., 2013). Indeed, blockade of SK channels with apamin increases the gain of the F-I relationship in spinal MNs (Zhang and Krnjević, 1987). How do Adrb2/Adrb3 receptors act on the SK channels? SK channels display constitutive trafficking, and PKA activation was shown to decrease the surface expression of SK channels through three phosphorylation sites (Ser568, Ser569 and Ser570 at the carboxyl-terminal region) (Ren et al., 2006). In the present work, we showed that an intracellular injection of cAMP-SP (a cAMP agonist) also increases the F-I gain relationship, similarly to the systemic delivery of Adrb2/Adrb3 agonists. Furthermore, we also showed that the pharmacological stimulation of Adrb2/Adrb3 Gs-coupled receptors effectively engages the cAMP/PKA pathway, since the neuromodulatory effects disappeared when PKA was inhibited by H89. We then speculate that the pharmacological stimulation of Adrb2/Adrb3 receptors indirectly removes SK channels from the plasma membrane of MNs through the activation of the cAMP/PKA pathway. A similar scenario was previously demonstrated at excitatory synapses from lateral amygdala pyramidal neurons (Faber et al., 2008). This would result in a decrease of the SK conductance, contributing to increase the F-I gain in the primary range, as observed in our experiments.

### 3.3. Chronic β-adrenergic stimulation results in homeostatic mechanisms that cancel physiological neuromodulation

Prolonged treatment with Adrb2/Adrb3 agonists of either WT or SOD1 animals results, in our experiments, in the loss of their neuromodulatory effects on MN electrophysiological properties. This homeostatic response may be caused by several mechanisms at transcriptional, post-transcriptional and post-translational levels. 1) We show that the *Adrb2*/*Adrb3* mRNA is downregulated upon chronic pharmacological stimulation of the Adrb2/Adrb3 receptors establishing a negative feedback loop. Notably, heterologous downregulation of other Gs-coupled GPCRs is observed upon Adrb2/Adrb3 agonists treatment (or chemogenetic stimulation of Gs signaling). Our findings are in agreement with the downregulation of adrenergic receptors mRNA in multiple organs upon chronic adrenergic stimulation (Mak et al., 1995; Lefkowitz et al., 2000; Milano et al., 2018) through mechanisms involving transcriptional regulation and mRNA stability (Danner et al., 1998), as well as with extensive heteronomous downregulation of Gs-coupled GPCRs. 2) A single injection of Adrb2/Adrb3 agonists rapidly and substantially modulates the expression of many channel-encoding genes in MNs, often in directions that oppose the effects of Adrb2/Adrb3 agonists on increasing MN excitability and firing. In particular, *Scn1a* (NaV1.1, sodium current) is strongly downregulated while *Kcnq3* (K_V_7.3) and *Kcnq5* (K_v_7.5), which mediate the M-current, are upregulated (although not *Kcnq2*) in both WT and SOD1 MNs contributing to reduce MN excitability (Sharples et al., 2023; Schroeder et al., 2000). On the same line Adrb2/Adrb3 agonists downregulate *Ano6* (encoding Tmem16f channels) counteracting the recruitment current decrease in both WT and SOD1 MNs (Soulard et al., 2020). Furthermore, while Adrb2/Adrb3 agonists decrease the AHP half-decay time, *Kcnn2* (SK2) is significantly upregulated in WT MNs (although not in SOD1 MNs), resulting in larger SK2 conductance that may increase the AHP decay time and counteract the F-I gain increase. While these transcriptional changes may only partially translate into protein-level changes within the time frame of the acute experiments (up to 3 h), they would most likely contribute to the homeostatic feedback after the chronic pharmacological treatment (10 days). 3) Post-translational mechanisms may also contribute to stabilizing β-adrenergic signaling against persistent stimulation. Upon activation, phosphorylation of the cytoplasmic tail of adrenergic receptors results in their interaction with β-arrestin adaptors that mediate their endosomal internalization and thereby signal desensitization (Hausdorff et al., 1991; Tran et al., 2004; Wachter and Gilbert, 2012) in a timeframe of minutes to hours. Moreover, persistent stimulation leads to the phosphorylation of the receptors by β-adrenergic receptor kinases (BARKs) triggering a switch in the affinity from Gs to Gi and the reversal of the Adenylyl Cyclase activation (Wang et al., 2017). These post-translational homeostatic mechanisms extend to other convergent receptors through heterologous desensitization mechanisms involving PKA phosphorylation (Benovic et al., 1985; Gainetdinov et al., 2004). To sum up, our findings show that, both healthy and ALS MNs display substantial homeostatic loops regulating PKA signaling and excitability responses to GPCR stimulation. Furthermore, the homeostatic regulation of the β-adrenergic neuromodulation upon prolonged agonist administration is the same in MNs from SOD1 and WT mice indicating that MNs are still displaying a proper gain in the feedback loops and a meaningful homeostatic regulation of excitability at a presymptomatic disease stage.

### 3.4. Study limitations

A few limitations should be considered within the context of the present work. First, the administration of the adrenergic agonist was performed intravenously in the acute electrophysiological experiments, but intraperitoneally for the chronic experiments. Indeed, a repeated i.v. injection was considered to impose an excessive burden on animals in chronic experiments, whereas using IP injections in the acute electrophysiological experiment would have negated the precise timing of the adrenergic activation. Extrapolating from existing literature (Yi et al., 2017; Al Shoyaib et al., 2019), we can speculate that i.v. may result in higher peak concentration and faster time-to-peak than i.p.; this may affect the saturation of adrenergic receptors and their inactivation kinetics, with i.p. more prone to induce inactivation and downregulation. This faster receptor saturation allowed us to precisely determine the beginning of the effects of β2/β3 agonists on MN excitability during acute experiments. However, over several hours, cumulative doses are not strongly affected by the route of administration (Yi et al., 2017; Al Shoyaib et al., 2019). Thus, the overall congruence of the two experimental designs may be affected but not negated by the irreducible necessity to use two administration routes. Second, the reciprocal experiment to Adrb2/3 activation by agonists, *i.e.* the use of β-adrenergic antagonists, could not be performed since the use of Adrb antagonists is complicated by the need to employ an adrenergic receptor α2 agonist in the electrophysiological procedure (hence, reducing adrenergic tone already to a minimum); in order to determine the role of Adrb at baseline, further experiments on mice in which the β-adrenergic receptors are specifically knocked-out on spinal motoneurons are needed. Third, although we showed that the cAMP-PKA pathway is a major signal transduction pathway of Adrb2/3, since the increase of the FI gain in WT and SOD1 MNs was prevented by the H89 PKA inhibitor, other signaling takes place through Gi or Erk pathway (Shenoy et al., 2006; O’Hayre et al., 2017) and directly through cAMP-sensitive channels (Martín et al., 2020). Adrb2/3 agonists elicited a differential modulation of Gin plateau/Gin ramp and Vthreshold for firing in WT and SOD1 MNs. This could be partially accounted for by the lower PKA activity basal level in SOD1 MNs (see Fig. 2 A), but we cannot exclude a contribution of the above-mentioned non-PKA pathways. Fourth, the DREADD-Gs system reproduces the cAMP-PKA signaling of the Adrb2/3, but not necessarily all the signaling, since it was originally derived from a muscarinic receptor to which the Gs-protein-interacting loops from a turkey adrenergic receptor were spliced (Roth, 2016); although DREADD-Gs and Adrb stimulation show some similarities in their adaptation effect, possible divergences may be attributed to non-PKA-dependent signaling (Shenoy et al., 2006; O’Hayre et al., 2017; Martín et al., 2020).

## 4. Conclusions

Our findings demonstrate that MNs are responsive to neuromodulatory signals not only from α- but also from β-adrenergic receptors, hinting at an integration of these two distinct adrenergic signaling cascades with each other and possibly with other neuromodulators. We demonstrate that homeostatic processes, at receptor and ion-channel levels, are set in motion by β-adrenergic stimulation aimed at restoring an excitability set-point both in normal and disease conditions. The function of the β-adrenergic neuromodulation remains to be clarified. In particular, we do not know whether it cooperates with the classical α-adrenergic neuromodulation. Finally, our work shows that the homeostasis of the β-adrenergic neuromodulation is not dysregulated in MNs from ALS mice at an early presymptomatic stage, suggesting that the β-adrenergic system is unlikely to contribute to the early pathophysiological mechanisms of ALS.

## 5. Materials and methods

### 5.1. Animals

All experiments were performed on the transgenic mouse line B6SJL-Tg(SOD1*G93A) 1Gur/J pursued via The Jackson Laboratory (#002726, Gurney et al., 1994). SOD1*G93A hemizygotes (here referred to as SOD1) develop a phenotype resembling human ALS as a consequence of the high number of transgene copies. Only males were used in Ulm (histology and transcriptomics experiments) in accordance with literature characterizing disease staging in this mouse strain (Gurney et al., 1994; Saxena et al., 2013; Ouali Alami et al., 2018; Bączyk et al., 2020), animals aged P40-P45 were deemed presymptomatic. Animal experiments from Ulm were conducted according to institutional guidelines (Tierforschungszentrum, Universität Ulm, Germany) under the approval of the Regierungspräsidium Tübingen with license nr. 1390 (untreated) and 1440 (drug-treated animals).

Animal experiments from Paris were approved by the Paris Descartes University ethics committee (CEEA34) and authorized by the French ministry for higher education and research (authorization number APAFIS#16338–2018052100307589). Animal experiments from Poznań were approved by the local ethical committee (16/2021). For electrophysiological experiments in Paris and in Poznań, both male and female mice were recorded between the ages of P45-P59.

Mice were grouped ≤ 5 with access to food and water *ad libitum* and housed in M2 long cages in an open shelving system (Ulm, Poznań) or disposable and ventilated cages (Paris) under a 12 h/12 h light/dark cycle with 40÷60% relative humidity. Upon routine tests assessing motor impairment, individuals reaching end-stage (on average >P110-P120) were euthanized complying with the aforementioned directives. In Paris, SOD1 mice were euthanized no later than P90.

For electrophysiological experiments B6SJL-Tg(SOD1*G93A)1Gur/J males were crossed with B6SJL/J females, and offspring carriers of the mutated SOD1 gene were selected for experimental groups (SOD1), with non-transgenic littermates serving as wild type (WT) control animals. For chemogenetics experiments (Regierungspräsidium Tübingen, license nr. 1404), females from the B6;129S6-ChAT tm2(cre)Lowl /J line (The Jackson Laboratory, stock #006410; Rossi et al., 2011) were crossed with SOD1 males; only F1 SOD1;ChAT-Cre males were used for intraspinal injections of AAV9 viruses enabling Cre-dependent expression of the Gs-DREADD construct in ChAT+ cells (viruses were produced as in Commisso et al., 2018).

### 5.2. Surgical procedures for electrophysiological experiments

In Paris and Poznań, electrophysiological experiments were carried out with the same protocol. Fifteen minutes before anesthesia, atropine (0.20 mg/kg; Aguettant or Polfa) and methylprednisolone (0.05 mg; Solu-Medrol; Pfizer) were given subcutaneously to prevent salivation and edema, respectively. Anesthesia was induced with an intraperitoneal (i.p.) injection of Fentanyl 0.025 mg/kg, Midazolam 7.5 mg/kg and Medetomidine 0.5 mg/kg. The heart rate was monitored with an EKG, and the central temperature was kept around 37°C using an infrared heating lamp and an electric blanket. Then, the mouse was artificially ventilated with pure oxygen (SAR-1000 ventilator; CWE) through a cannula inserted in the trachea. The ventilator settings were adjusted to maintain the end-tidal CO_2_ level at ~4% (Micro-Capstar; CWE). Two catheters were introduced in the external jugular veins. The first one was used to deliver supplemental doses of anesthesia whenever necessary (usually every 20–30 min) by i.v. injection (10% of the dose used for anesthesia induction). The adequacy of anesthesia was assessed by lack of noxious reflexes and stability of the heart rate (usually 400–500 bpm) and end-tidal pCO_2_. The other catheter was used to slowly inject (50 μL/h) a 4% glucose solution containing NaHCO_3_ (1%) and gelatin (15%; Voluven; Fresenius Kabi or Tetraspan; Braun) to maintain the physiological parameters and to inject the tested treatment. The tibial and common peroneal branches of the sciatic nerve were dissected and mounted on a bipolar electrode for stimulation. The vertebral column was immobilized with two pairs of horizontal bars (Cunningham Spinal Adaptor; Stoelting) applied on the Th12 and L2 vertebral bodies, and the L3–L4 spinal segments were exposed by a laminectomy at the Th13–L1 level. The exposed tissues from the hindlimb and spinal cord were covered with pools of mineral oil. When the surgery was completed, the animal was paralyzed with vecuronium or pancuronium with a bolus of 0.5 mg/kg as needed. Additional doses of anesthetic were then provided at the same frequency as before the paralysis, and adequacy of anesthesia was assessed by stability of the heart rate and pCO_2_. At the end of the experiment, animals were euthanized with a lethal i.p. injection of pentobarbital (Exagon or Morbital; 80 mg).

### 5.3. Stimulation and intracellular recordings

Intracellular recordings of MNs were performed with micropipettes (tip diameter, 1.0–1.5μm) filled with 2 M K-acetate (resistance ~25MΩ). Recordings were made using an Axoclamp 2B (Paris) or Axoclamp 900 A (Poznań) amplifier (Molecular Devices) connected to a Power1401 interface (sampling rate 20 kHz) and using Spike2 software (CED). After impalement, identification of MNs relied on the observation of antidromic action potentials in response to the electrical stimulation of the tibial and common peroneal nerves together, a few millimeters proximal to the popliteal fossa. All MNs retained for analysis had a resting membrane potential more hyperpolarized than −50 mV and an overshooting action potential > 65 mV. For more details on the analysis, see Manuel et al. (2009).

Briefly, the input conductance was determined from the I-V relationship, which was plotted from the peak (Gin peak) and plateau (Gin plateau) responses to a series of small-amplitude square current pulses (−2 to +2 nA, 500 ms). Discharge properties were investigated using slow triangular ramps of currents (0.5–2 nA/s). The discharge frequency-current relationship (F-I) was determined in the primary range by plotting the instantaneous firing frequency against the current intensity. We estimated the AHP half-decay time relaxation time constant (a parameter close to the relaxation time constant, Manuel et al., 2009) on spike triggered averaging of the 3–5 spikes with the longest interspike intervals in the subprimary range, as in Fig. 4 G and 4I. The AHP half-decay time is the time between the peak amplitude and half the peak amplitude, with the peak being measured between the voltage before spike and the most hyperpolarized voltage of the AHP. However, the AHP relaxation time constant is measured by fitting an exponential on the tail of the AHP, which is highly sensitive to the noise on the tail. Therefore, the AHP half-decay time is easier to estimate using a 3–5 spike triggered average. We verified that in the selected interspikes intervals the AHP fully relaxed; the half-decay time was on average 4.5 times shorter than the average of interspike intervals. With this method, the distribution and mean of the AHP half-decay time are similar as the ones found in Manuel et al. (2009). The input conductance was also determined from the early linear voltage response (<2 nA) to a slow ramp of current (Gin ramp). All recordings were performed in discontinuous current clamp mode (8 kHz). All care was taken to compensate for the microelectrode resistance and capacitance.

### 5.4. PKA activation experiments

The procedures for surgery, MN stimulation and recording were performed as described above. To measure the impact of PKA activation on MN intrinsic excitability, the recording microelectrode was filled with a mixture of 2 M K-acetate, and 4 mM cAMP analogue (S)-adenosine, cyclic 39,59- (hydrogenphosphorothioate) triethylammonium (cAMP-SP; Sigma). Upon penetration of a MN, series of square current pulses and slow ramp of currents were repeated in successive 3–5 min intervals for at least 15 min, until the properties of the MN stabilized. Then, the iontophoretic injection of cAMP-SP was performed through the microelectrode (500 ms pulses of −2 to −4 nA, repeated at 1 Hz, total normalized electrical load injected 2600 ± 600 nA.sec/μS). We adjusted the injected current to the MN input conductance (normalized electrical load) because we noticed that MNs with higher conductance needed more current injection to observe the same effects as in MNs with smaller conductance. Immediately after, the square current pulses and slow ramp of currents were repeated with the same parameters as before the activation of the cAMP/PKA pathway. Data were excluded when the resting membrane potential changed by more than ±5 mV or the bridge balance changed by more than ± 20%, to ensure that the changes in excitability are not due to an insufficient stability of the MN or the microelectrode.

### 5.5. Surgical procedures for intraspinal AAV injections

Intraspinal surgery and AAV injection procedures were performed as in Bączyk et al. (2020). In short, P18 to P21 mice first received a subcutaneous dose of buprenorphine (0.1 mg/kg) and meloxicam (1 mg/kg) and from then onwards they were kept anesthetized onto the stereotaxic frame via continuous administration of 2% isoflurane in O_2_ at 0.8 L/min O_2_ flow rate. After making a small incision on the skin, the subcutaneous fascia was removed and the paraspinal muscles were gently pushed sideways and blunt-dissected. The underlying spinal cord was exposed by conducting laminectomy on T12 and removing the vertebral bone flap. Using a Picospritzer microfluidic device, 1 μL of a 1:1 mixture of virus suspension and 1% Fast Green dye in PBS+ + was injected with a pulled glass capillary at the following stereotaxic coordinates: y = +0.25 mm with respect to the dorsal artery; 0.4 mm depth below the dorsal surface of the spinal cord. Before withdrawing it, the capillary was left in place for 10 min to allow the viral suspension to diffuse locally and to avoid viral backflow. Over the next 3 days, mice were treated with meloxicam (daily, 1 mg/kg) and buprenorphine (twice a day, 0.1 mg/kg) and monitored for post-operative complications.

### 5.6. Drug treatments

The Adrb ligands selected for acute or chronic (10 days-long) intraperitoneal (i.p.) treatment were amibegron (A; Cayman Chemicals 11954), an Adrb3 receptor agonist, and formoterol fumarate (F; LKT Laboratories F5868), an Adrb2 receptor agonist. All drugs were diluted in a mixture of 5% dimethyl sulfoxide (DMSO) 5% Tween-80 5% polyethylenglycole-400 (Roth) in saline to achieve the designated daily dosage (AF, 10 mg/kg A and 0.3 mg/kg F). Intracardiac perfusions took place 3 h after the latest injection.

For electrophysiological experiments, A and F stock solutions were separately diluted in DMSO (Sigma) and stored at −20°C until the day of the experiment. The day of the experiment, stock solutions were diluted in saline (B.Braun) and mixed together to obtain the desired dose (A: 3 mg/kg and F: 0.3 mg/kg). The AF cocktail was injected i.v. after at least one MN was recorded. Just after the injection of AF, an increased heart rate was clearly observable on the EKG and started to decrease after 3 h. Because β-adrenergic receptors are expressed in the heart and are known to increase the heart rate (Lohse et al., 2003), we used the heart rate as an indicator of an ongoing activation of β-adrenergic receptors. Therefore, MNs were recorded and kept for analysis for up to 3 h after the injection of AF.

We selected H89 (Tocris 2910) as a PKA inhibitor, which was also diluted in DMSO (Sigma) and stored at −20°C until the day of the experiment. The day of the experiment, stock solution was diluted in saline to obtain 2 doses of 10 mg/kg of H89 injected i.p. before recording the first MNs and later, during the experiment, at the same time as the AF cocktail.

For chemogenetics experiments, mice injected with Gs-DREADD-expressing AAV9 were administered the Gs-DREADD agonist clozapine N-oxide (CNO) between P35 and P45 i.p. route twice per day at 5 mg/kg, with an 8 h interval between consecutive treatments. Drugs were solubilized in DMSO and diluted in saline (2.5% DMSO in the final mixture). Intracardiac perfusions took place 3 h after the last injection.

### 5.7. Laser capture microdissection and RT-qPCR

Mice received terminal anesthesia by i.p. administration of ketamine (100 mg/kg; WDT) and xylazine (16 mg/kg; Bayer) and were perfused with RNAse-free ice-cold PBS for 2 min at 7 mL/min flow rate. The lumbar portion of the spinal cord was quickly dissected out, OCT embedded (Sakura) and stored at −80°C. 12μm-thick cryosections were converted onto RNAse-free polyethylene terephthalate membrane (PET) slides (Zeiss 415190–9051–000) once these had been coated with poly-L-Lysine (Sigma Aldrich).

Firstly, a fixation step was performed in −20°C-cold 70% ethanol in RNAse-free H_2_O (DEPC ddH_2_O: 1% diethylpyrocarbonate (Roth) in ddH_2_O). Next, the slides were stained for 1 min in 4°C-cold 1% cresyl violet (WALDECK) in 50% ethanol-DEPC ddH_2_O and finally washed for 1 min in 4°C-cold 70% and then 100% ethanol-DEPC ddH_2_O. 30 MNs per group were microdissected into 500 μL adhesive caps (Zeiss) with a laser microdissection system (Palm MicroBeam, Zeiss). MNs were lysed by pipetting 21 μL of a mixture comprising 30 μL 10X RT buffer, 3 μL RNAse OUT (Invitrogen^™^ SuperScript^™^ III Reverse Transcriptase kit, 11904018) and 0.3 μL 1% NP-40 in ddH_2_O directly onto the adhesive cap. After vortexing upside-down for 30 s and incubating for 20 min at 42°C, samples were frozen at −80°C overnight.

Reverse transcription and RNA digestion were carried out with the cDNA synthesis kit as per manufacturer’s instructions. RNA content was determined via qPCR with a Roche LC480 cycler using 2 μL cDNA. PCR cycles were set as follows: 2 min at 50°C, 10 min at 95°C (initial denaturation); 50 cycles comprising 15 s at 95°C (denaturation) and 1 min at 60°C for annealing and elongation. The relative quantification of the transcripts of interest relied on the ΔCt method upon normalization against *Gapdh* housekeeping gene; the sequences of the primers used in this study, designed with NCBI Primer designing tool (Primer-BLAST, Ye et al., 2012) are reported in Supplemental Table 1.

### 5.8. Histology

Terminally anesthetized P45 animals were perfused first with 50 mL ice-cold PBS (prepared in house) and 50 mL 4% paraformaldehyde (PFA; Sigma-Aldrich) in PBS at an approx. 7 mL/min flow speed. Overnight fixation in 4% PFA in PBS preceded incubation in 30% sucrose (Roth) in PBS and OCT embedding. Samples were equilibrated overnight at −20°C before being sequentially cut into 15μm-thick sections.

*In situ* hybridization (ISH) experiments were carried out with the RNAscope Fluorescent multiplex reagent kit v1 (Advanced Cell Diagnostics, ACD; Wang et al., 2012) and the following ACD probes: Mmp9-C1 (315941), Adrb1-C3 (449761), Adora2b-C3 (445281), Drd5-C3 (494411), Adrb2-C3 (449771), Adrb3-O1-C3 (502581). The manufacturer protocol for fixed frozen tissue was followed with minor deviations (for detailed procedures, refer to olde Heuvel et al., 2019). For the quantification of *Adrb1*–*3* mRNA, because of logistical limitations, two sets of experiments were performed (two distinct sets of animals); in order to keep an internal quality measure, the *Adrb1* mRNA was performed in both sets (set 1: *Adrb1* + *Adrb2*; set 2: *Adrb1* + *Adrb3*). To prevent a selection bias, we have merged the two *Adrb1* datasets (which displayed a similar trend with each other); thus *Adrb1* appears to be determined on a larger N than *Adrb2* or *Adrb3*.

Subsequently, sections were co-immunostained to identify α-MNs; to prevent ISH signal disruption, the slides were always incubated in a humid chamber and protected from light. In short, sections were washed twice in 1X Wash Buffer for 10 min at room temperature (RT), incubated for 1 h at RT in blocking buffer (BB: 10% Bovine Serum Albumin 0.3% Triton^™^X-100 (Sigma-Aldrich) in 1X PBS) and finally with primary antibodies overnight at 4°C. In this manuscript primary antibodies (anti-VAChT, Synaptic Systems, 139105; anti-mCherry, NanoTag Biotechnologies, N0404-AT565; anti-p62, Abcam, ab56416) were prediluted 1:250 in BB. Three 30 min washes in 0.1% Triton^™^X-100 in 1X PBS (PBS-T) at RT preceded incubation with pre-diluted secondary antibodies (1:250 in BB) for 2 h at RT. All secondary antibodies (Sigma, SAB4600468; Thermo Fisher Scientific, A10037) used for co-immunostaining were applied at 1:250 dilution. For free-floating immunostaining experiments, sections were incubated in BB for 2 h and with primary antibodies (anti-misfSOD1 1:1000, Médimabs MM-0070; anti-VAChT 1:500, Synaptic Systems, 139105; anti-phospho-PKA substrate 1:100, CST 9621) for 48 h; secondary antibodies (Invitrogen, A21202; Biotium, 20171; Invitrogen, A32790) were applied at 1:500 dilution.

### 5.9. Imaging

All experiments comprising an ISH step were imaged using a confocal laser scanning microscope (Carl Zeiss LSM710) equipped with a Plan-Apochromat 63X oil objective (NA 1.40). Images were acquired with a 1024 × 1024 frame size at 12-*bit* depth (0.132μm/px resolution in *xy*; 0.57μm voxel depth) preferentially at the surface of the section where probe penetration was highest and thus consistent between groups. The same image acquisition settings were employed to image misfSOD1 accumulation and phospho-PKA epitopes, but with a 20X objective (NA 0.8). Only ventrolateral MNs were investigated in this study.

### 5.10. Image processing and quantification

Maximum intensity projections of confocal *z*-stacks and in general all downstream processing and image analysis steps were instead performed in *Fiji* (Schindelin et al., 2012). *ImageJ* custom macros enabled semi-automated identification, drawing and ID assignment of MN *somata* according to VAChT signal. Regions of interest were then extracted from ISH channels (upon a background subtraction step, *radius* 5) and their size was measured retaining information on the receptor probe, the experimental group and the MN ID in their name tag. A similar approach was adopted to measure cytoplasmic PKA phospho-epitopes and whole-cell misfSOD1 accumulation as mean fluorescence intensity per MN cross section area. ISH signal was quantified as spot density per μm^2^ by means of custom Jython and *ImageJ* macro scripts exploiting the difference of Gaussian detector shipped with Trackmate plugin (Tinevez et al., 2017); peak size and intensity threshold were set upon visual inspection of the experimental group given the slight variability and efficiency of probe penetration in different batches.

### 5.11. Data analysis and statistics

All RT-qPCR data were first normalized against *Gapdh* mRNA levels and, subsequently, against the control group (WT group for the LCM-based GPCR screening; WT vehicle for acute drug treatment experiments; SOD1 vehicle for chronic administration experiments). To test whether MN mRNA expression differed between WT and SOD1 mice, a *t*-test for each gene was computed. Heatmaps of LCM-based data were generated with the *ggplot2* R package.

All ISH as well as p62 and misfSOD1 immunohistochemistry data were first normalized against the control group for a meaningful comparison of the experimental sets; a quality-control step was further included to determine MN cross-section areas and assess whether the sampling was uniform or otherwise introducing unintended bias. Upon visual inspection of receptorome scatterplots (*Mmp9 vs.* receptor mRNA, *data not shown*), for all screenings a threshold of 0.3 spots per μm^2^ was chosen in order to subdivide MNs into *Mmp9*+ and *Mmp9*− categories. *Adrb2* and *Adrb3* expression data were handled with the *tidyverse* R package. The heatmap was computed after gene-wise z score data normalization in R.

For GPCR ISH screening, to test whether MN mRNA expression differed between WT and SOD1 mice and whether this effect was different between *Mmp9*− and *Mmp9*+ MNs, a generalized linear mixed model was calculated for each gene. Fixed effects for genotype (WT or SOD1) and motoneuron type (*Mmp9*− or *Mmp9*+), their interaction effect, a random per-animal offset, and a full-factorial dispersion model were included. A gaussian error distribution with an identity-link was used for *Adrb1* and *Adrb2* expression data, whereas for *Adrb3* data a Gamma distribution with a log-link was necessary to meet model assumptions.

For immunohistology of RRXpS/T, to test whether the acute treatment (vehicle or agonist) affected PKA-phosphorylation in MNs and whether this effect differed between WT and SOD1 mice, a linear mixed model with genotype, treatment and their interaction as fixed effects and a per-animal random offset was calculated.

For immediate early genes and ion channels RT-qPCR, to test if the acute treatment (vehicle or agonist) affected MN mRNA expression and whether this effect differed between WT and SOD1 mice, a linear model with genotype and treatment as well as their interaction were calculated for each gene. The heatmap was computed after gene-wise z score data normalization in R, whereas the PCA plot was generated with Clustvis software (Metsalu and Vilo, 2015).

For GPCR ISH upon chemogenetic treatment, to test whether the chronic DREADD(Gs) activation affected MN transcription differed in infected (Gs+) or in non-infected (Gs−) MNs, a generalized linear mixed model with MN type (Gs+ or Gs−) as a fixed and dispersion effect, a peranimal random offset, and a Gamma error-distribution with a log-link function were calculated for each gene.

For GPCR ISH upon adrenergic agonist treatment, to test whether the chronic treatment (vehicle or agonist) affected MN transcription, a generalized linear mixed model with treatment as a fixed and dispersion effect, a per-animal random offset, and a log-link function were calculated for each gene. For *Adora2b*, a Gamma error distribution was used.

For misfSOD1 immunohistology, to test whether the chronic treatment affected misfSOD1 levels, a generalized linear mixed model with treatment as a fixed and dispersion effect, a per-animal random offset, and a log-link function was calculated.

For electrophysiology, to test if the treatment affected intrinsic neuronal properties and whether this effect differed between WT and SOD1 mice, we calculated linear mixed models for each intrinsic parameter. Fixed effects for treatment (acute: before or after; chronic: vehicle or agonist) and genotype (WT or SOD1) as well as their interaction effect were included. To account for between animal variance, a random per-animal offset was introduced. Backwards elimination was performed on the random effect, *i.e.*, the random offset was only included if it significantly contributed to goodness-of-fit of the model.

Data obtained from the same MNs recorded before and after the iontophoretic injection of cAMP-SP were compared using a paired *t*-test except for maximum firing frequency, for which a Wilcoxon paired test was used.

Model assumptions were confirmed both by visually inspecting the quantile-quantile plots and histograms, and by performing distribution, dispersion, outlier, homogeneity of variance, and quantile deviation tests using the DHARMa R package (Hartig, 2022). If model assumptions were violated, following strategies were employed. Data points were identified as outliers, if the absolute value of their residual was greater than 2 standard deviations, and removed. In case of non-normality of residuals, generalized-linear (mixed) models with link function and error distributions that did not violate model assumptions were chosen. A full-factorial dispersion model was fit in case of presence of heteroscedasticity. Degrees of freedom of linear mixed models were adjusted using the Kenward-Roger method. Linear models were fit using lm, linear mixed models using *lmer* of the *lme4* R-package (Bates et al., 2015), and generalized linear (mixed) models using *glmmTMB* (a Template Model Builder interface for R; Brooks et al., 2017). Planned *post-hoc* tests were performed to test if there was a significant treatment effect within WT or SOD1 mice. Throughout, an alpha-error of *p* < 0.05 was regarded as significant.

## Supplementary Material

1

2

3

## Data Availability

Data will be made available on request.

## Appendix A. Supporting information

Supplementary data associated with this article can be found in the online version at doi:10.1016/j.pneurobio.2026.102905.

## References

[R1] Al Shoyaib A, Archie SR, Karamyan VT, 2019. Intraperitoneal route of drug administration: should it be used in experimental animal studies? Pharm. Res 37, 12. 10.1007/s11095-019-2745-x. 31873819 PMC7412579

[R2] Bączyk M, Alami NO, Delestrée N, Martinot C, Tang L, Commisso B, Bayer D, Doisne N, Frankel W, Manuel M, Roselli F, Zytnicki D, 2020. Synaptic restoration by cAMP/PKA drives activity-dependent neuroprotection to motoneurons in ALS. J. Exp. Med 217. 10.1084/jem.20191734. PMC7398175 32484501

[R3] Bączyk M, Manuel M, Roselli F, Zytnicki D, 2022. From physiological properties to selective vulnerability of motor units in amyotrophic lateral sclerosis. Adv. Neurobiol 28, 375–394. 10.1007/978-3-031-07167-6_15. 36066833

[R4] Bates D, Mächler M, Bolker B, Walker S, 2015. Fitting linear mixed-effects models using lme4. J. Stat. Softw 67, 1–48. 10.18637/jss.v067.i01.

[R5] Benovic JL, Pike LJ, Cerione RA, Staniszewski C, Yoshimasa T, Codina J, Caron MG, Lefkowitz RJ, 1985. Phosphorylation of the mammalian beta-adrenergic receptor by cyclic AMP-dependent protein kinase. Regulation of the rate of receptor phosphorylation and dephosphorylation by agonist occupancy and effects on coupling of the receptor to the stimulatory guanine nucleotide regulatory protein. J. Biol. Chem 260, 7094–7101. 2987243

[R6] Bouvier M, Collins S, O’Dowd BF, Campbell PT, de Blasi A, Kobilka BK, MacGregor C, Irons GP, Caron MG, Lefkowitz RJ, 1989. Two distinct pathways for cAMP-mediated down-regulation of the beta 2-adrenergic receptor. Phosphorylation of the receptor and regulation of its mRNA level. J. Biol. Chem 264, 16786–16792. 2476447

[R7] Branchereau P, Martin E, Allain A-E, Cazenave W, Supiot L, Hodeib F, Laupénie A, Dalvi U, Zhu H, Cattaert D, 2019. Relaxation of synaptic inhibitory events as a compensatory mechanism in fetal SOD spinal motor networks. Elife 8. 10.7554/eLife.51402. PMC6974356 31868588

[R8] Bras H, Jankowska E, Noga B, Skoog B, 1990. Comparison of effects of various types of NA and 5-HT agonists on transmission from group II muscle afferents in the cat. Eur. J. Neurosci 2, 1029–1039. 10.1111/j.14609568.1990.tb00015.x. 12106064

[R9] Brooks ME, Kristensen K, Benthem KJ, Magnusson A, Berg CW, Nielsen A, Skaug H, Mächler M, Bolker B, 2017. glmmTMB balances speed and flexibility among packages for zero-inflated generalized linear mixed modeling. R. J 10.32614/RJ-2017-066.

[R10] Brownstone RM, Lancelin C, 2018. Escape from homeostasis: spinal microcircuits and progression of amyotrophic lateral sclerosis. J. Neurophysiol 119, 1782–1794. 10.1152/jn.00331.2017. 29384454 PMC6008087

[R11] Carr DB, Day M, Cantrell AR, Held J, Scheuer T, Catterall WA, Surmeier DJ, 2003. Transmitter modulation of slow, activity-dependent alterations in sodium channel availability endows neurons with a novel form of cellular plasticity. Neuron 39, 793–806. 10.1016/s0896-6273(03)00531-2. 12948446

[R12] Cavarsan CF, Steele PR, Henry LT, Reedich EJ, McCane LM, LaPre KJ, Puritz AC, Manuel M, Katenka N, Quinlan KA, 2023. Inhibitory interneurons show early dysfunction in a SOD1 mouse model of amyotrophic lateral sclerosis. J. Physiol 601, 647–667. 10.1113/JP284192. 36515374 PMC9898203

[R13] Commisso B, Ding L, Varadi K, Gorges M, Bayer D, Boeckers TM, Ludolph AC, Kassubek J, Müller OJ, Roselli F, 2018. Stage-dependent remodeling of projections to motor cortex in ALS mouse model revealed by a new variant retrograde-AAV9. Elife 7. 10.7554/eLife.36892. PMC6125125 30136928

[R14] Danner S, Frank M, Lohse MJ, 1998. Agonist regulation of human beta2-adrenergic receptor mRNA stability occurs via a specific AU-rich element. J. Biol. Chem 273, 3223–3229. 10.1074/jbc.273.6.3223. 9452435

[R15] Deardorff AS, Romer SH, Deng Z, Bullinger KL, Nardelli P, Cope TC, Fyffe REW, 2013. Expression of postsynaptic Ca2+-activated K+ (SK) channels at C-bouton synapses in mammalian lumbar -motoneurons. J. Physiol 591, 875–897. 10.1113/jphysiol.2012.240879. 23129791 PMC3591704

[R16] Delestrée N, Manuel M, Iglesias C, Elbasiouny SM, Heckman CJ, Zytnicki D, 2014. Adult spinal motoneurones are not hyperexcitable in a mouse model of inherited amyotrophic lateral sclerosis. J. Physiol 592, 1687–1703. 10.1113/jphysiol.2013.265843. 24445319 PMC3979619

[R17] Faber ESL, Delaney AJ, Power JM, Sedlak PL, Crane JW, Sah P, 2008. Modulation of SK channel trafficking by beta adrenoceptors enhances excitatory synaptic transmission and plasticity in the amygdala. J. Neurosci 28, 10803–10813. 10.1523/JNEUROSCI.1796-08.2008. 18945888 PMC6671376

[R18] Gainetdinov RR, Premont RT, Bohn LM, Lefkowitz RJ, Caron MG, 2004. Desensitization of G protein-coupled receptors and neuronal functions. Annu Rev. Neurosci 27, 107–144. 10.1146/annurev.neuro.27.070203.144206. 15217328

[R19] Gonzalez-Hernandez AJ, Munguba H, Levitz J, 2024. Emerging modes of regulation of neuromodulatory G protein-coupled receptors. Trends Neurosci. 47, 635–650. 10.1016/j.tins.2024.05.008. 38862331 PMC11324403

[R20] Gurney ME, Pu H, Chiu AY, Dal Canto MC, Polchow CY, Alexander DD, Caliendo J, Hentati A, Kwon YW, Deng HX, 1994. Motor neuron degeneration in mice that express a human Cu,Zn superoxide dismutase mutation. Science 264, 1772–1775. 10.1126/science.8209258. 8209258

[R21] Hammar I, Bannatyne BA, Maxwell DJ, Edgley SA, Jankowska E, 2004. The actions of monoamines and distribution of noradrenergic and serotoninergic contacts on different subpopulations of commissural interneurons in the cat spinal cord. Eur. J. Neurosci 19, 1305–1316. 10.1111/j.1460-9568.2004.03239.x. 15016088 PMC1971244

[R22] Hartig F, 2022. DHARMa: residual diagnostics for hierarchical (multi-level / mixed) regression models. R. Package Version 0 (4), 6. 10.32614/CRAN.package.DHARMa.

[R23] Hausdorff WP, Campbell PT, Ostrowski J, Yu SS, Caron MG, Lefkowitz RJ, 1991. A small region of the beta-adrenergic receptor is selectively involved in its rapid regulation. Proc. Natl. Acad. Sci. USA 88, 2979–2983. 10.1073/pnas.88.8.2979. 1849641 PMC51367

[R24] Henry AM, Hohmann JG, 2012. High-resolution gene expression atlases for adult and developing mouse brain and spinal cord. Mamm. Genome 23, 539–549. 10.1007/s00335-012-9406-2. 22832508

[R25] Huang B, Li Y, Cheng D, He G, Liu X, Ma L, 2018. β-Arrestin-biased β-adrenergic signaling promotes extinction learning of cocaine reward memory. Sci. Signal 11. 10.1126/scisignal.aam5402. 29317519

[R26] Iglesias C, Meunier C, Manuel M, Timofeeva Y, Delestrée N, Zytnicki D, 2011. Mixed mode oscillations in mouse spinal motoneurons arise from a low excitability state. J. Neurosci 31 (15), 5829–5840. 10.1523/JNEUROSCI.6363-10.2011. 21490224 PMC6622841

[R27] Ito M, Oshima T, 1965. Electrical behaviour of the motoneurone membrane during intracellularly applied current steps. J. Physiol 180, 607–635. 10.1113/jphysiol.1965.sp007720. 5846796 PMC1357406

[R28] Jensen DB, Kadlecova M, Allodi I, Meehan CF, 2020. Spinal motoneurones are intrinsically more responsive in the adult G93A SOD1 mouse model of amyotrophic lateral sclerosis. J. Physiol 598, 4385–4403. 10.1113/JP280097. 32716521

[R29] Jordan LM, Liu J, Hedlund PB, Akay T, Pearson KG, 2008. Descending command systems for the initiation of locomotion in mammals. Brain Res. Rev 57, 183–191. 10.1016/j.brainresrev.2007.07.019. 17928060

[R30] Kanning KC, Kaplan A, Henderson CE, 2010. Motor neuron diversity in development and disease. Annu Rev. Neurosci 33, 409–440. 10.1146/annurev.neuro.051508.135722. 20367447

[R31] Kaplan A, Spiller KJ, Towne C, Kanning KC, Choe GT, Geber A, Akay T, Aebischer P, Henderson CE, 2014. Neuronal matrix metalloproteinase-9 is a determinant of selective neurodegeneration. Neuron 81, 333–348. 10.1016/j.neuron.2013.12.009. 24462097 PMC6015650

[R32] Kernell D, 1999. Repetitive impulse firing in motoneurons: facts and perspectives. Prog. Brain Res 123, 31–37. 10.1016/s0079-6123(08)62841-1. 10635701

[R33] Kuo S-W, Binder MD, Heckman CJ, 2020. Excessive homeostatic gain in spinal motoneurons in a mouse model of amyotrophic lateral sclerosis. Sci. Rep 10, 9049. 10.1038/s41598-020-65685-8. 32493926 PMC7271238

[R34] Lefkowitz RJ, Rockman HA, Koch WJ, 2000. Catecholamines, cardiac beta-adrenergic receptors, and heart failure. Circulation 101, 1634–1637. 10.1161/01.cir.101.14.1634. 10758041

[R35] Leroy F, Lamotte d’Incamps B, Imhoff-Manuel RD, Zytnicki D, 2014. Early intrinsic hyperexcitability does not contribute to motoneuron degeneration in amyotrophic lateral sclerosis. Elife 3. 10.7554/eLife.04046. PMC4227046 25313866

[R36] Lochner A, Moolman JA, 2006. The many faces of H89: a review. Cardiovasc Drug Rev. 24, 261–274. 10.1111/j.1527-3466.2006.00261.x. 17214602

[R37] Lohse MJ, Engelhardt S, Eschenhagen T, 2003. What is the role of beta-adrenergic signaling in heart failure? Circ. Res 93, 896–906. 10.1161/01.RES.0000102042.83024.CA. 14615493

[R38] Luo H, Marron Fernandez de Velasco E, Wickman K, 2022. Neuronal G protein-gated K+ channels. Am. J. Physiol. Cell Physiol 323, C439–C460. 10.1152/ajpcell.00102.2022. 35704701 PMC9362898

[R39] Maaliki D, Jaffa AA, Nasser S, Sahebkar A, Eid AH, 2024. Adrenoceptor desensitization: current understanding of mechanisms. Pharm. Rev 76, 358–387. 10.1124/pharmrev.123.000831. 38697858 PMC12164723

[R40] Mak JC, Nishikawa M, Shirasaki H, Miyayasu K, Barnes PJ, 1995. Protective effects of a glucocorticoid on downregulation of pulmonary beta 2-adrenergic receptors in vivo. J. Clin. Invest 96, 99–106. 10.1172/JCI118084. 7615841 PMC185177

[R41] Manuel M, Iglesias C, Donnet M, Leroy F, Heckman CJ, Zytnicki D, 2009. Fast kinetics, high-frequency oscillations, and subprimary firing range in adult mouse spinal motoneurons. J. Neurosci 29, 11246–11256. 10.1523/JNEUROSCI.3260-09.2009. 19741131 PMC2785440

[R42] Manuel M, Meunier C, Donnet M, Zytnicki D, 2006. The afterhyperpolarization conductance exerts the same control over the gain and variability of motoneurone firing in anaesthetized cats. J. Physiol 576, 873–886. 10.1113/jphysiol.2006.117002. 16931549 PMC1890407

[R43] Maratta R, Fenrich KK, Zhao E, Neuber-Hess MS, Rose PK, 2015. Distribution and density of contacts from noradrenergic and serotonergic boutons on the dendrites of neck flexor motoneurons in the adult cat. J. Comp. Neurol 523, 1701–1716. 10.1002/cne.23765. 25728799

[R44] Marder E, Goaillard J-M, 2006. Variability, compensation and homeostasis in neuron and network function. Nat. Rev. Neurosci 7, 563–574. 10.1038/nrn1949. 16791145

[R45] Martin E, Cazenave W, Cattaert D, Branchereau P, 2013. Embryonic alteration of motoneuronal morphology induces hyperexcitability in the mouse model of amyotrophic lateral sclerosis. Neurobiol. Dis 54, 116–126. 10.1016/j.nbd.2013.02.011. 23466698

[R46] Martín R, García-Font N, Suárez-Pinilla AS, Bartolomé-Martín D, Ferrero JJ, Luján R, Torres M, Sánchez-Prieto J, 2020. β-adrenergic receptors/epac signaling increases the size of the readily releasable pool of synaptic vesicles required for parallel fiber LTP. J. Neurosci 40, 8604–8617. 10.1523/JNEUROSCI.0716-20.2020. 33046543 PMC7643300

[R47] Martínez-Silva M. de L., Imhoff-Manuel RD, Sharma A, Heckman CJ, Shneider NA, Roselli F, Zytnicki D, Manuel M, 2018. Hypoexcitability precedes denervation in the large fast-contracting motor units in two unrelated mouse models of ALS. Elife 7, e30955. 10.7554/eLife.30955. 29580378 PMC5922970

[R48] McLarnon JG, 1995. Potassium currents in motoneurones. Prog. Neurobiol 47, 513–531. 10.1016/0301-0082(95)00032-1. 8787033

[R49] Metsalu T, Vilo J, 2015. ClustVis: a web tool for visualizing clustering of multivariate data using principal component analysis and heatmap. Nucleic Acids Res. 43, W566–W570. 10.1093/nar/gkv468. 25969447 PMC4489295

[R50] Meunier C, Borejsza K, 2005. How membrane properties shape the discharge of motoneurons: a detailed analytical study. Neural Comput. 17, 2383–2420. 10.1162/0899766054796923. 16156933

[R51] Milano S, Gerbino A, Schena G, Carmosino M, Svelto M, Procino G, 2018. Human β3-adrenoreceptor is resistant to agonist-induced desensitization in renal epithelial cells. Cell Physiol. Biochem 48, 847–862. 10.1159/000491916. 30032151

[R52] Montague SJ, Fenrich KK, Mayer-Macaulay C, Maratta R, Neuber-Hess MS, Rose PK, 2013. Nonuniform distribution of contacts from noradrenergic and serotonergic boutons on the dendrites of cat splenius motoneurons. J. Comp. Neurol 521, 638–656. 10.1002/cne.23196. 22821606

[R53] Nascimento F, ×zyurt MG, Halablab K, Bhumbra GS, Caron G, Bączyk M, Zytnicki D, Manuel M, Roselli F, Brownstone R, Beato M, 2024. Spinal microcircuits go through multiphasic homeostatic compensations in a mouse model of motoneuron degeneration. bioRxivorg 10.1101/2024.04.10.588918. PMC11847574 39656589

[R54] Nicholas AP, Hökfelt T, Pieribone VA, 1996. The distribution and significance of CNS adrenoceptors examined with in situ hybridization. Trends Pharm. Sci 17, 245–255. 10.1016/0165-6147(96)10022-5. 8756183

[R55] O’Hayre M, Eichel K, Avino S, Zhao X, Steffen DJ, Feng X, Kawakami K, Aoki J, Messer K, Sunahara R, Inoue A, 2017. Genetic evidence that β-arrestins are dispensable for the initiation of β2-adrenergic receptor signaling to ERK. Sci. Signal 10, eaal3395. 10.1126/scisignal.aal3395. 28634209 PMC5751434

[R56] Okeke K, Angers S, Bouvier M, Michel MC, 2019. Agonist-induced desensitisation of β3 -adrenoceptors: Where, when, and how? Br. J. Pharm 176, 2539–2558. 10.1111/bph.14633. PMC6592865 30809805

[R57] Heuvel F. olde, Holl S, Chandrasekar A, Li Z, Wang Y, Rehman R, Förstner P, Sinske D, Palmer A, Wiesner D, Ludolph A, Huber-Lang M, Relja B, Wirth T, Röszer T, Baumann B, Boeckers T, Knöll B, Roselli F, 2019. STAT6 mediates the effect of ethanol on neuroinflammatory response in TBI. Brain Behav. Immun 81, 228–246. 10.1016/j.bbi.2019.06.019. 31207335

[R58] Ouali Alami N, Schurr C, Heuvel F. Olde, Tang L, Li Q, Tasdogan A, Kimbara A, Nettekoven M, Ottaviani G, Raposo C, Röver S, Rogers-Evans M, Rothenhäusler B, Ullmer C, Fingerle J, Grether U, Knuesel I, Boeckers TM, Ludolph A, Wirth T, Roselli F, Baumann B, 2018. NF-κB activation in astrocytes drives a stage-specific beneficial neuroimmunological response in ALS. EMBO J. 37. 10.15252/embj.201798697. PMC6092622 29875132

[R59] Pun S, Santos AF, Saxena S, Xu L, Caroni P, 2006. Selective vulnerability and pruning of phasic motoneuron axons in motoneuron disease alleviated by CNTF. Nat. Neurosci 9, 408–419. 10.1038/nn1653. 16474388

[R60] Quinlan KA, Schuster JE, Fu R, Siddique T, Heckman CJ, 2011. Altered postnatal maturation of electrical properties in spinal motoneurons in a mouse model of amyotrophic lateral sclerosis: Accelerated maturation of motoneurons in amyotrophic lateral sclerosis. J. Physiol 589, 2245–2260. 10.1113/jphysiol.2010.200659. 21486770 PMC3098701

[R61] Rekling JC, Funk GD, Bayliss DA, Dong XW, Feldman JL, 2000. Synaptic control of motoneuronal excitability. Physiol. Rev 80, 767–852. 10.1152/physrev.2000.80.2.767. 10747207 PMC4764886

[R62] Ren Y, Barnwell LF, Alexander JC, Lubin FD, Adelman JP, Pfaffinger PJ, Schrader LA, Anderson AE, 2006. Regulation of surface localization of the small conductance Ca2+-activated potassium channel, Sk2, through direct phosphorylation by cAMP-dependent protein kinase. J. Biol. Chem 281, 11769–11779. 10.1074/jbc.M513125200. 16513649

[R63] Rossi J, Balthasar N, Olson D, Scott M, Berglund E, Lee CE, Choi MJ, Lauzon D, Lowell BB, Elmquist JK, 2011. Melanocortin-4 receptors expressed by cholinergic neurons regulate energy balance and glucose homeostasis. Cell Metab. 13, 195–204. 10.1016/j.cmet.2011.01.010. 21284986 PMC3033043

[R64] Roth BL, 2016. DREADDs for neuroscientists. Neuron 89, 683–694. 10.1016/j.neuron.2016.01.040. 26889809 PMC4759656

[R65] Saxena S, Roselli F, Singh K, Leptien K, Julien J-P, Gros-Louis F, Caroni P, 2013. Neuroprotection through excitability and mTOR required in ALS motoneurons to delay disease and extend survival. Neuron 80, 80–96. 10.1016/j.neuron.2013.07.027. 24094105

[R66] Schindelin J, Arganda-Carreras I, Frise E, Kaynig V, Longair M, Pietzsch T, Preibisch S, Rueden C, Saalfeld S, Schmid B, Tinevez J-Y, White DJ, Hartenstein V, Eliceiri K, Tomancak P, Cardona A, 2012. Fiji: an open-source platform for biological-image analysis. Nat. Methods 9, 676–682. 10.1038/nmeth.2019. 22743772 PMC3855844

[R67] Schroeder BC, Hechenberger M, Weinreich F, Kubisch C, Jentsch TJ, 2000. KCNQ5, a novel potassium channel broadly expressed in the brain, mediates M-type currents. J. Biol. Chem 275, 24089–24095. 10.1074/jbc.M003245200. 10816588

[R68] Sharp FR, Sagar SM, 1994. Alterations in gene expression as an index of neuronal injury: heat shock and the immediate early gene response. Neurotoxicology 15, 51–59. 8090362

[R69] Sharples SA, Broadhead MJ, Gray JA, Miles GB, 2023. M-type potassium currents differentially affect activation of motoneuron subtypes and tune recruitment gain. J. Physiol 601, 5751–5775. 10.1113/JP285348. 37988235

[R70] Shenoy SK, Drake MT, Nelson CD, Houtz DA, Xiao K, Madabushi S, Reiter E, Premont RT, Lichtarge O, Lefkowitz RJ, 2006. β-Arrestin-dependent, G protein-independent ERK1/2 activation by the β2 adrenergic receptor. J. Biol. Chem 281, 1261–1273. 10.1074/jbc.M506576200. 16280323

[R71] Smith CC, Brownstone RM, 2022. Electrical properties of adult mammalian motoneurons. Adv. Neurobiol 28, 191–232. 10.1007/978-3-031-07167-6_9. 36066827

[R72] Song J, Dikwella N, Sinske D, Roselli F, Knöll B, 2023. SRF deletion results in earlier disease onset in a mouse model of amyotrophic lateral sclerosis. JCI Insight 8. 10.1172/jci.insight.167694. PMC10445689 37339001

[R73] Soulard C, Salsac C, Mouzat K, Hilaire C, Roussel J, Mezghrani A, Lumbroso S, Raoul C, Scamps F, 2020. Spinal motoneuron TMEM16F acts at C-boutons to modulate motor resistance and contributes to ALS pathogenesis. Cell Rep. 30, 2581–2593.e7. 10.1016/j.celrep.2020.02.001. 32101737

[R74] Takahashi T, 1990. Inward rectification in neonatal rat spinal motoneurones. J. Physiol 423, 47–62. 10.1113/jphysiol.1990.sp018010. 2388157 PMC1189745

[R75] Tartas M, Morin F, Barrière G, Goillandeau M, Lacaille J-C, Cazalets J-R, Bertrand SS, 2010. Noradrenergic modulation of intrinsic and synaptic properties of lumbar motoneurons in the neonatal rat spinal cord. Front Neural Circuits 4, 4. 10.3389/neuro.04.004.2010. 20300468 PMC2839852

[R76] Tinevez J-Y, Perry N, Schindelin J, Hoopes GM, Reynolds GD, Laplantine E, Bednarek SY, Shorte SL, Eliceiri KW, 2017. TrackMate: an open and extensible platform for single-particle tracking. Methods 115, 80–90. 10.1016/j.ymeth.2016.09.016. 27713081

[R77] Tran TM, Friedman J, Qunaibi E, Baameur F, Moore RH, Clark RB, 2004. Characterization of agonist stimulation of cAMP-dependent protein kinase and G protein-coupled receptor kinase phosphorylation of the beta2-adrenergic receptor using phosphoserine-specific antibodies. Mol. Pharm 65, 196–206. 10.1124/mol.65.1.196. 14722251

[R78] Turrigiano GG, 1999. Homeostatic plasticity in neuronal networks: the more things change, the more they stay the same. Trends Neurosci. 22, 221–227. 10.1016/s0166-2236(98)01341-1. 10322495

[R79] Wachter SB, Gilbert EM, 2012. Beta-adrenergic receptors, from their discovery and characterization through their manipulation to beneficial clinical application. Cardiology 122, 104–112. 10.1159/000339271. 22759389

[R80] Wang F, Flanagan J, Su N, Wang L-C, Bui S, Nielson A, Wu X, Vo H-T, Ma X-J, Luo Y, 2012. RNAscope: a novel in situ RNA analysis platform for formalin-fixed, paraffin-embedded tissues. J. Mol. Diagn 14, 22–29. 10.1016/j.jmoldx.2011.08.002. 22166544 PMC3338343

[R81] Wang J, Hanada K, Staus DP, Makara MA, Dahal GR, Chen Q, Ahles A, Engelhardt S, Rockman HA, 2017. Gαi is required for carvedilol-induced β1 adrenergic receptor β-arrestin biased signaling. Nat. Commun 8, 1706. 10.1038/s41467-017-01855-z. 29167435 PMC5700200

[R82] Ye J, Coulouris G, Zaretskaya I, Cutcutache I, Rozen S, Madden TL, 2012. Primer-BLAST: a tool to design target-specific primers for polymerase chain reaction. BMC Bioinforma. 13, 134. 10.1186/1471-2105-13-134. PMC3412702 22708584

[R83] Yi B, Jahangir A, Evans AK, Briggs D, Ravina K, Ernest J, Farimani AB, Sun W, Rajadas J, Green M, Feinberg EN, Pande VS, Shamloo M, 2017. Discovery of novel brain permeable and G protein-biased beta-1 adrenergic receptor partial agonists for the treatment of neurocognitive disorders. PLoS One 12, e0180319. 10.1371/journal.pone.0180319. 28746336 PMC5529018

[R84] Zhang L, Krnjević K, 1987. Apamin depresses selectively the after-hyperpolarization of cat spinal motoneurons. Neurosci. Lett 74, 58–62. 10.1016/0304-3940(87)90051-6. 2436107

