# Supplementary material for: Spinal motoneuron excitability is homeostatically regulated through β-adrenergic neuromodulation in wild-type and presymptomatic SOD1 mice
Source: Prog Neurobiol. Author manuscript; Available in PMC 2026 Jun 23. (PMC13288352; doi:10.1016/j.pneurobio.2026.102905)
Supplement: 1 [file NIHMS2181274-supplement-1.docx]

**Supplemental Table 1 | qPCR primer list.**

| **Housekeeping gene** | **Forward primer sequence (5’-3’)** | **Reverse primer sequence (5’-3’)** |
| --- | --- | --- |
| *Gapdh* | TGGATCTGACGTGCCGC | TGCCTGCTTCACCACCTTC |

| **Negative control gene** | **Forward primer sequence (5’-3’)** | **Reverse primer sequence (5’-3’)** |
| --- | --- | --- |
| *Gfp* | GAAGCGCGATCACATGGT | CCATGCCGAGAGTGATCC |

| **GPCR-encoding genes** | **Forward primer sequence (5’-3’)** | **Reverse primer sequence (5’-3’)** |
| --- | --- | --- |
| *Adcyap1r1* | TATGGACTTCAAGCACCGGC | TCTTGCTCAGGATGGACAGC |
| *Adora2a* | GTTAGGTAGGCAGAGGGACAGG | CTGCGATTGCTTCCCTTCTCTG |
| *Adora2b* | GGAACCGAGACTTCCGCTAC | GACTGAGAGTAGACTGCGCC |
| *Adrb1* | CTACAACGACCCCAAGTGCT | ACGTAGAAGGAGACGACGGA |
| *Adrb2* | TACACAGGGGAGCCAAACAC | TCAACGCTAAGGCTAGGCAC |
| *Adrb3* | CAGGCTCTGTGTCTCTGGTTA | GAGGAGACAGGGATGAAACCTC |
| *Agtr1a* | CTTAGGGTTGGAACCTGCGG | TCATCCAGTCCCTCCCAACT |
| *Bdkrb1* | CCGCTACAGGTTGCTGGTAT | TTGACGGAACGCAGAAGGAA |
| *Cckar* | ACTGCCAAGTCCACGTTCAA | TCATCTGGGGCGTTCCAAAA |
| *Chrm2* | ACTGCCATTGCGGCTTTCTA | TATTCTGCTCTTGCTCGCCC |
| *Chrm4* | GCCTCTGGCTAGTTCCGCC | TCGCCATGCTGAACCCAAC |
| *Drd2* | GACACCACTCAAGGGCAACT | ATCCATTCTCCGCCTGTTCA |
| *Drd5* | CGAACCTACGCCATCTCCTC | GCGCGTGTAGGTCACTATCA |
| *Gabbr2* | ACAGGCGATTCCAGTTCACA | CGTAGGCGGTGGTTTTCTGA |
| *Gpr3* | ATCTACGCCTTTCGCAACCA | CGGGACCGGAATGGAATCTT |
| *Gpr65* | CATGGGCTACGCAATACCCT | TGTTTTCCGTGGCTTGGTTG |
| *Gpr68* | ACGATACCAGCCCAAGTGTG | CACCTTAACCAGTCCTCTGGC |
| *Grm4* | TACCAGTACCAACGTCGCAA | GCATCCGCTCTATTCTGAGGT |
| *Grm8* | TGTGCTCCTAACGGGGATTT | GATGATTGTGTCAGGTGCCG |
| *Hcar1* | AGTGTGAAGGAAACCGTGGG | CGCTTTTCTCAGCCATGCAA |
| *Hcar2* | GCGGCCATCATTTCTTGCTT | GCCTCGCCATTTTTGGTCAT |
| *Hrh3* | TTAGAGCATCAACCCGGCAG | CACTCCAGTTCCACCAACGA |
| *Htr7* | GTGGTCAAAATGGGAAACGGA | CCATTCTGCCTCACGGGGTA |
| *Lpar2* | GGCAGATGACTTGACTTCGC | GCCTCCCTGAATGTTTGCTC |
| *Oprd1* | TGGATGCTTTTGGGGTTCCT | AAACAAAGGGTCTCGGTGCT |
| *Oprl1* | TCCTCAGGCACACCAAGATG | GAAGGGCAGTGTCAGCAAGA |
| *P2ry12* | AACGCCAGTGTCATTTGCTG | TCTCCTTTTATTCTTGCACTGTGAC |
| *Pthr1* | AGCGAGTGCCTCAAGTTCAT | TCCCACGGTGTAGATCATGC |
| *S1pr5* | AACTCGCTGCTGAATCCCAT | GGAGGAGTCTTGGTTGCAGG |
| *Tacr1* | AGGTGTCTGGGGGTTTCTTTA | CCTAGAAGTGACAGGTGACCA |

| **Immediate-early genes** | **Forward primer sequence (5’-3’)** | **Reverse primer sequence (5’-3’)** |
| --- | --- | --- |
| *ΔFosB* | AGGCAGAGCTGGAGTCGGAGAT | GCCGAGGACTTGAACTTCACTCG |
| *c-Fos* | CCTGCCCCTTCTCAACGAC | GCTCCACGTTGCTGATGCT |
| *Egr1* | GCCGAGCGAACAACCCTAT | TCCACCATCGCCTTCTCATT |
| *NPas4* | GCTATACTCAGAAGGTCCAGAAGGC | TCAGAGAATGAGGGTAGCACAGC |

| **Ion channel-encoding genes** | **Channel name** | **Forward primer sequence (5’-3’)** | **Reverse primer sequence (5’-3’)** |
| --- | --- | --- | --- |
| *Cacna1d* (isof.1) | Ca_V_1.3 | GCTCGGTGGCTGTATTTTCAA | CCGTGCTTTCTACCGCACTT |
| *Cacna2d3* | Cacn α2δ3 | GCAGATCGCAGGAAGCTTTG | ACGGGAGATTTCCGCTCATC |
| *Hcn1* | Hcn1 | CGTGAAGCATGACCGAGAGA | GTAGACTGGCGGAGATTGGG |
| *Hcn2* | Hcn2 | CATCCACACCAAAGCCATGC | CCCGCCTCCTAAGCTACCTA |
| *Kcna1* | K_V_1.1 | TGCTGTGTGTCGCTCAATCT | TCTCCGAACTGGACACTTGC |
| *Kcna2* | K_V_1.2 | TACCCATCTGCAAGGGCAACG | CGACTTGAGGAGGAGAGTGGA |
| *Kcnab1* | Kcnaβ1 / K_V_β1 | TCTTGGACTGGTCCCCTACC | AGATTCCCCTACCCCAGCAT |
| *Kcnb1* | K_V_2.1 | GAGAGGGCGTGGCTAAGAAG | GCCCTCTTGGTCCATTTCCA |
| *Kcnj14* | K_IR_2.4 | GCCGAGGACAGACCTGAACAC | ACTGGGGGTTCCTCTGCTCA |
| *Kcnn3* | K_Ca_2.3 / SK3 | ATCCACCGTCATCCTGCTTG | GTAGGTCATGGCTATCCGCC |
| *Kcnn2* | K_Ca_2.2 / SK2 | ACAAGGCGTCGCTGTATTCT | CTGTATTTCCCTGGCGTGGT |
| *Kcnq2* | K_V_7.2 | GCCATTTTGTACGTGCCCTT | TAGAAGACAGCGTCGTGTGC |
| *Kcnq3* | K_V_7.3 | AGTCTTGCTTCCCTGGTGATTC | TCGTCCTGCATTTGGCTGATA |
| *Kcnq5* | K_V_7.5 | GCAGCCACCAGACTAAAGGA | CTGCCGCTTCCAATTCCAAA |
| *Kcnt2* | K_Na_1.2 / Slick | CTGTGCACTTAAAAGCAATACAGT | AGCATTTTCCACATCCATGACT |
| *Scn1a* | Na_V_1.1 | TACAGAAGCAGACCGTAGGC | TGTGATTAGCATCATTTTGGGCT |
| *Scn8a* | Na_V_1.6 | CCTTCTTACGAGACCCGTGG | ACCCTGAAAGTGCGTAGAGC |
| *Ano6* | Ano6 / Tmem16f | TGGAACCCTGATCTTCGCTG | TTGCTGTAGCTCAACGGTGT |
| *Trpm5* | Trpm5 | GAATGGGGACTACAGAGGCTG | CGAATGTTTCCTGTGGAGGC |


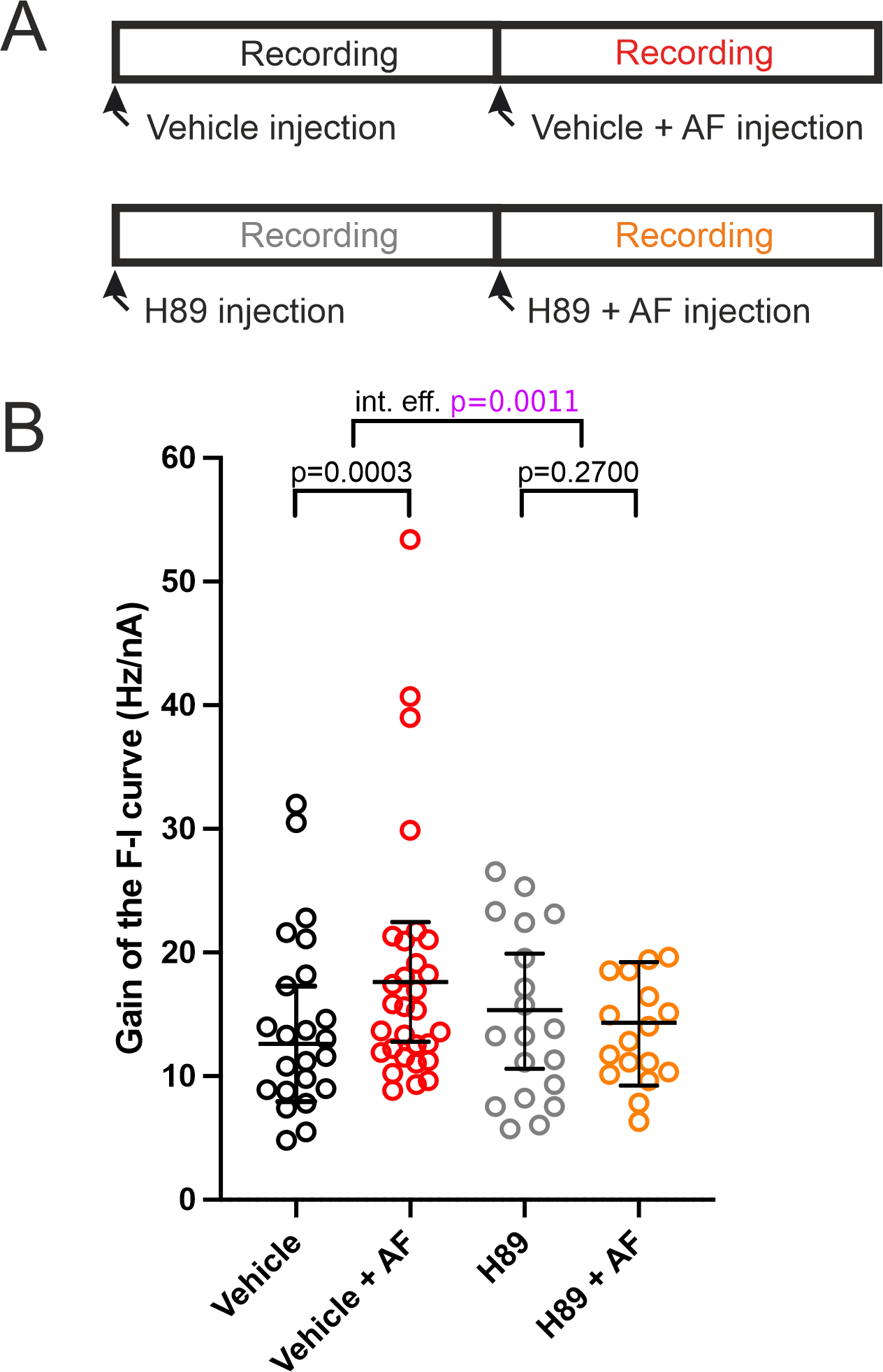


**Supplemental Figure 1 | H89 abolishes the effects of Adrb2/Adrb3 agonists on the firing gain of MNs in SOD1 mice. A)** Electrophysiological experiments were performed before and after the acute injection of AF in the absence or in the presence of the PKA blocker, H89. **B)** The increase in the firing gain of MNs induced by the acute injection of AF is abolished in the presence of H89. Each point represents one MN and the mean ± 95% confidence intervals are shown. Significances on top bars illustrate interaction effects (magenta). *Post-hoc* significances are shown for Vehicle before AF *vs.* Vehicle after AF (treatment effect in the presence of Vehicle), H89 before AF *vs.* H89 after AF (treatment effect in the presence of H89). Amibegron + Formoterol (AF). N = 6 SOD1 mice for Vehicle before and after AF and N = 6 SOD1 mice for H89 before and after AF.

**
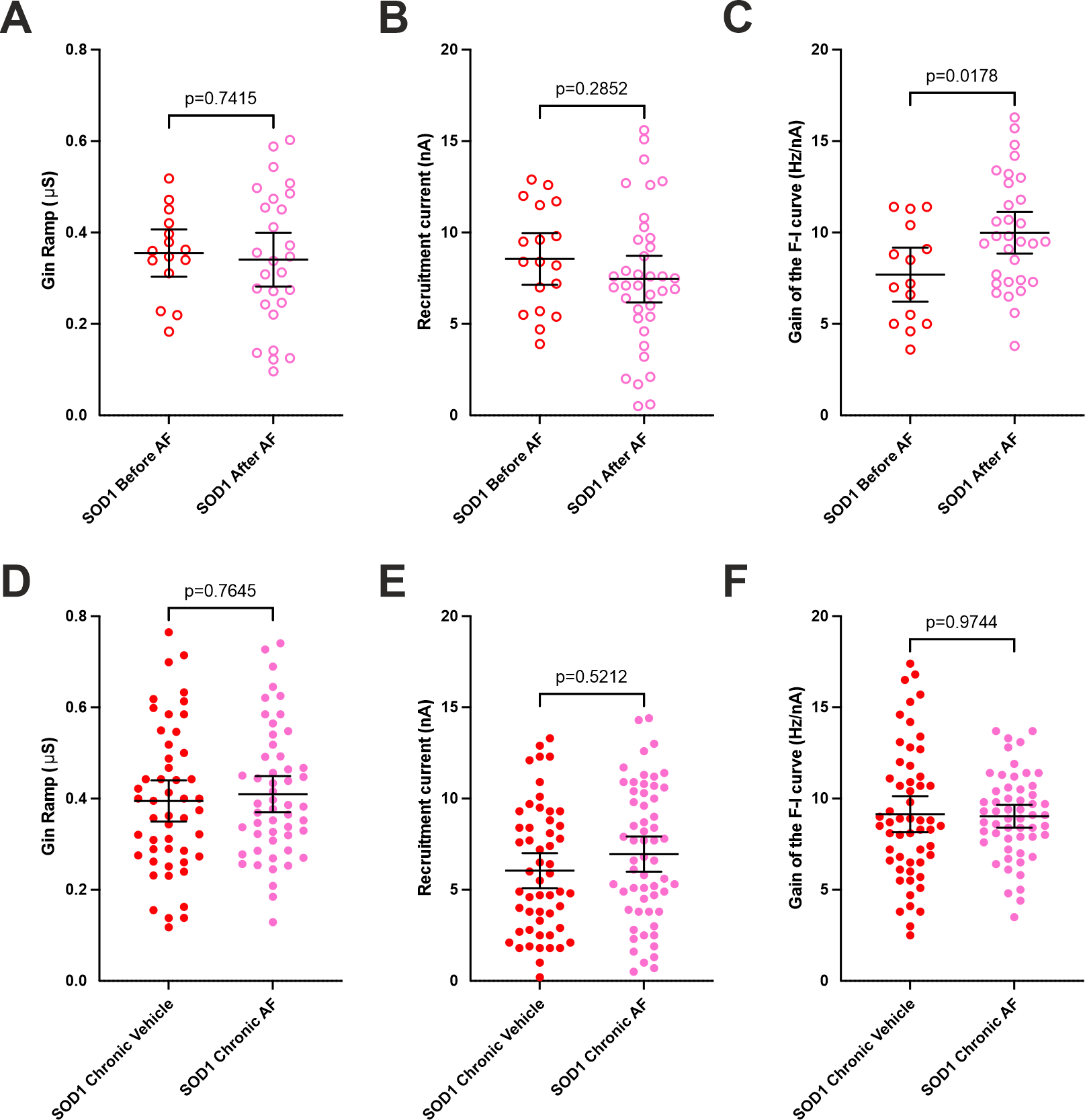
**

**Supplemental Figure 2 | Replication of electrophysiological findings upon acute and prolonged delivery of Adrb2/Adrb3 agonists on a different cohort of presymptomatic SOD1 mice. A-H)** Electrophysiological properties were obtained from slow ramps of current, as in Figures 4 and 7. **A-C)** Effect of the acute treatment on ramp input conductance **(A)**, recruitment current **(B)**, gain of the F-I relationship **(C),** in MNs from SOD1 mice. **D-F)** Effect of the chronic treatment on ramp input conductance **(D)**, recruitment current **(E)**, gain of the F-I relationship **(F),** in MNs from SOD1 mice. In all graphs, each point represents one MN and the mean ± 95% confidence intervals are shown. N = 7 Acute SOD1 mice and N = 11 Chronic SOD1 mice.
