# Supplementary material for: Spinal motoneuron excitability is homeostatically regulated through β-adrenergic neuromodulation in wild-type and presymptomatic SOD1 mice
Source: Prog Neurobiol. Author manuscript; Available in PMC 2026 Jun 23. (PMC13288352; doi:10.1016/j.pneurobio.2026.102905)
Supplement: 2 [file NIHMS2181274-supplement-2.pdf]

Peer Review Overview

Manuscript Title: "Spinal motoneuron excitability is homeostatically-regulated through β-adrenergic neuromodulation in wild-type and presymptomatic SOD1 mice"

Received	24-Jan-2025
1st Decision	27-Apr-2025
1st Revision Submitted	28-Oct-2025
2nd Decision	04-Jan-2026
2nd Revision Submitted	23-Jan-2026
Accepted	08-Mar-2026

1st Decision Letter

Dear Dr. Caron,

Thank you for submitting your manuscript to Progress in Neurobiology. We appreciate your patience during this unusually lengthy review procecss.

We have completed our evaluation of your manuscript. The reviewers recommend reconsideration of your manuscript following major revision. We invite you to resubmit your manuscript after addressing the comments below. Please resubmit your revised manuscript by **Jul 28, 2025**.

When revising your manuscript, please consider all issues mentioned in the reviewers' comments carefully: please outline every change made in response to their comments and provide suitable rebuttals for any comments not addressed. Please note that your revised submission may need to be re-reviewed.

To submit your revised manuscript, please log in as an author at https://www.editorialmanager.com/proneu/, and navigate to the "Submissions Needing Revision" folder.

Research Elements (optional)
This journal encourages you to share research objects - including your raw data, methods, protocols, software, hardware and more – which support your original research article in a Research Elements journal. Research Elements are open access, multidisciplinary, peer-reviewed journals which make the objects associated with your research more discoverable, trustworthy and promote replicability and reproducibility. As open access journals, there may be an Article Publishing Charge if your paper is accepted for publication. Find out more about the Research Elements journals at https://www.elsevier.com/authors/tools-and-resources/research-elements-journals?dgcid=ec_em_research_elements_email.

Progress in Neurobiology values your contribution and we look forward to receiving your revised manuscript.

Kind regards,

Jeannie Chin, PhD
Co-Editor-in-Chief
Progress in Neurobiology

Reviewer comments:

Reviewer #1: In this paper, the authors investigated homeostatic feedback mechanisms regulating motoneuron excitability in presymptomatic SOD1-G93A ALS mice. They found that β2 and β3 adrenergic receptors are expressed in spinal motor neurons of both WT and SOD1 mice at P45 and play a role in neuromodulation. Activation of these receptors influences motoneurons firing properties through ion channel transcriptional changes, leading to receptor downregulation as part of a homeostatic response. Importantly, they report that homeostatic feedback loops remain intact in presymptomatic SOD1 mice, suggesting that early-stage ALS does not disrupt these regulatory mechanisms.
This study provides valuable insights into adrenergic modulation of motoneuron excitability in presymptomatic ALS by contributing to the understanding of homeostatic mechanisms in early ALS pathology. The study is well-structured, with clear experimental design, making a significant contribution to the field of ALS research.
Major suggestions:
- ALS is a progressive neurodegenerative disease, and the presymptomatic phase may exhibit subtle changes that are not immediately detectable at P45. I would suggest that the authors consider additional time points beyond P45 to assess whether homeostatic feedback remains intact throughout the presymptomatic phase. It is possible that subtle dysregulations in homeostatic mechanisms may not be visible at P45 but could emerge later in the disease course, even before the onset of motor symptoms. Including earlier (e.g., P30) or later (e.g., P60) time points might provide a more comprehensive view of the dynamics of feedback regulation in SOD1 mice and strengthen the conclusions about the stability of homeostatic mechanisms.
-While assessing misfolded SOD1 levels is valuable, it may not be sufficient to conclusively claim no impact on motoneurons. Other measures of motoneuron integrity should be considered, such as for instance motoneurons count (e.g., ChAT, NeuN) and axonal degeneration assessment (e.g. NFL, SMI32) in spinal cord sections.
- I would suggest that the authors consider expanding the investigation to include the effects of β2 adrenergic receptor antagonists on motoneuron excitability, which could provide valuable insights into the mechanistic role of β2 receptors in ALS pathology.
-If homeostatic feedback remains intact at P45, how do the authors explain this finding with the progressive motoneuron dysfunction observed in ALS mice? Would disrupting these adrenergic feedback loops accelerate or slow disease progression? A discussion on the potential therapeutic implications of β-adrenergic modulation would strengthen the manuscript.

Reviewer #2: The study aims to investigate whether neuromodulation of motor neuron (MN) intrinsic properties is homeostatically regulated in presymptomatic adult SOD1 ALS mice compared to age-matched wild-type (WT) controls. Specifically, the researchers examine the expression and regulation of Gs-coupled β2- and β3-adrenergic receptors in MNs, assessing their role in modulating MN firing through the cAMP/PKA pathway. By analyzing receptor desensitization following chronic activation, the study seeks to determine whether homeostatic plasticity of β-adrenergic signaling occurs in ALS MNs compared to WT controls and whether this mechanism contributes to MN excitability regulation, potentially identifying therapeutic targets.

Although this is an original and innovative topic, a key concern is the limited evidence linking these findings to MN neuropathology and ALS disease.

Specific Comments:

1-The selective increase of Adrb1 mRNA exclusively in vulnerable (Mmp9+) MNs at the early presymptomatic stage suggests that β1-adrenergic receptors may play a crucial role in ALS pathogenesis. However, the study focuses solely on Adrb2/3, which remain unchanged and are assumed to contribute to the homeostatic regulation of MN intrinsic properties. This conceptual framework is a bit difficult to grasp and, in my opinion, deserves a clearer justification.

2-In Figure 2, the authors demonstrate that acute intraperitoneal (IP) injection of β2 and β3 agonists (AF) activates PKA signaling (phosphorylation) in SOD1 MNs but not in WT MNs three hours post-treatment. However, the same treatment induces immediate-early gene expression (cFos, FosB) in laser-captured MNs (LCM-MNs) of both WT and SOD1 mice, suggesting that these two events (PKA activation and altered gene expression) may not be strictly linked.

3-The electrophysiological analysis of MNs indicates that intravenous (IV) injection (not IP as above) of β2/β3 agonists enhances MN excitability by decreasing H-current in SOD1 MNs but not in WT MNs. Conversely, the treatment lowers the voltage threshold for firing in MNs of WT mice but not in SOD1 MNs. This suggest that ALS MNs do not fully respond to β2/β3 adrenergic signaling. Could this be due to the lower basal levels of PKA signalling in SOD1MNs compared to WT MNs , making them less responsive? This limitation raises concerns about the efficacy of β2/β3 agonists in modulating SOD1 MNs excitability.
Additionaly, the rationale for using IV instead of IP administration should be clarified.

4-To further evaluate whether the cAMP/PKA pathway mediates these effects, the authors intracellularly injected a cAMP analog into MNs and measured its impact on excitability. While this intervention mimics some effects of β2/β3 agonists, a more direct demonstration would require testing the effect of PKA inhibitors. Additionally, the authors did not assess beta-arrestin/ERK activation, leaving open the possibility of an alternative signaling pathway involvement. Therefore, their conclusions regarding cAMP/PKA signaling should be tempered accordingly.

Direct cAMP/PKA activation in MNs was also achieved using DREADD expression via AAV9 in SOD1/ChAT-Cre double-transgenic mice (Figure 8). Why the authors did not monitor these mice electrophysiologically? It would have been a validation and support of previous observations.

The study reports that a single dose of β2/β3 agonists significantly alters ion channel gene expression within three hours in both WT and SOD1 mice. If the treatment was administered via IP, the pharmacokinetics of IP versus IV routes should be discussed, as they could differentially impact MN excitability.

The prolonged delivery of β2/β3 agonists, administered via IP, was compared to the acute IV treatment. This may be a criticalt issue when comparing the responses. It is unclear whether the second experiment conducted by an independent group in another lab was done under the same conditions. This should be clarified.

Chronic β2/β3 agonist administration reduced β1-β3 receptor and Drd5 mRNA levels, whereas chronic chemogenetic PKA activation selectively reduced β1 mRNA. This discrepancy suggests the involvement of additional pathways in the long-term effects of β2/β3 receptor activation, which warrants further investigation.

Conclusion:
This study provides valuable insights into the modulation of MN excitability via β2/β3 adrenergic receptors and contributes to understanding the homeostatic feedback mechanisms involved. However, the absence of pathological and functional outcomes related to ALS, such as MN loss, morphological /histopathological MN damage, or even neuroinflammation reactivity in spinal cord , raises questions regarding the therapeutic relevance of these findings. Addressing these limitations would strengthen the study's impact and translational potential.

Reviewer #3: This is a groundbreaking study that demonstrates for the first time neuromodulation through beta2/3 adrenergic receptors impacts MN transcription and translation, including altered expression of voltage gated ion channels, dopaminergic and adrenergic receptors in the receptive MNs. This is novel for spinal MNs, as previous work has documented adrenergic effects in MNs via alpha and beta1 receptor activation. The authors further demonstrate that presymptomatic SOD1 MNs respond largely in a similar way to beta2/3 modulation as WT MNs.

This paper presents a lot of great results, but it is not quite organized in a way that makes sense. There seems to be a disconnect between what is anticipated to be a paper on mechanisms of pathology in ALS and what turns out to be more of a paper on regulation of neural excitability of MNs in a more general sense. It might benefit the authors to decide what the main thrust of this paper is. If it is the difference between WT and SOD1 MNs, then pursuing more complete results on the effects of the increased expression of beta1 adrenergic receptors in SOD1 MNs would be essential. What is the impact of the increased expression, if indeed the increase in adrb1 mRNA results in increased surface expression of

the receptors, and increased sensitivity to beta1 agonists in SOD1 MNs. Authors state that “We de-prioritized the β1 receptor because it was already affected by the disease, agonists do not cross the blood-brain barrier well and they produce substantial systemic effects.” De-prioritizing the beta1 receptor because it is affected by the disease does not really make sense if the paper’s focus is on disease mechanisms of ALS. It would possibly make sense if the receptor expression appeared to be down regulated in SOD1 MNs and they would likely be less sensitive to receptor activation but in fact they are likely more sensitive, not less. Alternatively, this could also be a great paper focused on a novel pathway for neuromodulation of MNs via beta2/3 receptor activation.

AHP measurements taken from firing on current ramps. Authors cite Manuel et al 2006 for the measurements, but the 2006 study used APs evoked by short stimuli delivered at 1-2Hz, while the present study averaged spikes from the sub primary zone of spikes fired in response to a current ramp. Based on the example in figure 4H-I the firing rate was ~15 Hz, which seems bit too fast to allow for an analysis of the AHP.
There was a decrease in the plateau conductance of SOD1 (but not WT) MNs in the presence of AF drugs to activate beta2/3 receptors. However, it appears that the “group” effect was not significant. Is that correct? Were persistent inward currents decreased by beta2/3 adrenergic receptor activation? Was conductance different between WT and SOD1 MNs?
How were the impacts of CNO on dopaminergic receptors controlled for in the DREADDs experiment?

Assessment of receptor expression. It not entirely clear if there is altered expression of beta adrenergic receptors in WT vs presymptomatic SOD1 MNs – transcriptomics indicates upregulation of Adrb1, downregulation of Adrb2 type beta adrenergic receptors, and no change in Adrb3 receptors, however in situ hybridization only supports the increase in Adrb1 / beta1 adrenergic receptor expression. Note that for Adrb1, where it appears there were 6-7 mice per group, results were significant, and non-sig for the other receptor subtypes where there were only 4 animals per group. It’s unfortunate that a specific assessment of the surface expression of the receptor proteins was not possible.

Language is unclear. For example, authors refer to beta2 and beta3 adrenergic receptors in the introduction but don’t mention which adrenergic receptors are encoded by which gene. The authors then switch to gene names in results section and it's not clear to readers which one is being studied. Further, rather than saying “acute” for the short term application of FA, it is referred to as “punctual,” which means “on time” and does not make sense in this context. In section 2.5, 19 ion channel genes are listed. It would be very helpful to also list which channels those genes encode.

Immediate early genes. In every gene assessed, the vehicle-treated SOD1MNs look similar to the AF-treated WT MNs, as if the immediate early genes are already activated in the SOD1 MNs. Is this a known early indicator of disease related cell stress? In contrast, why would the RRxpS/T levels be lower than normal?

Proofreading is needed throughout the manuscript

Line 272 mentions “full blown spikes” but are spikes not consistently all-or-none?
Labels for significance are confusing, including both asterisks and hashtags above each bracket. Please clarify when interaction is found, what variables they are referring to.
Figure 4B Voltage acceleration would be easier to see if a straight line was added, at the initial slope of the voltage increase.
Fig 4G Arrows are not really effective at showing the variability in firing rate - including the frequency plot above would be helpful here.
Legend for Figure 4 seems to list the panels out of sequential order.

1st Author Response Letter

To the attention of prof. Jeannie Chin and of the Editorial Board of Progress in Neurobiology

Hereby we submit to your attention for publication in Progress in Neurobiology a revised version of the manuscript entitled “Spinal motoneuron excitability is homeostatically-regulated through β-adrenergic neuromodulation in wild-type and presymptomatic SOD1 mice” by Antonucci et al.

We would like to thank the reviewers for their positive comments and their remarks that help us to improve our manuscript. We have addressed all issues raised by the reviewers, performed new experiments and modified, integrated and amended the manuscript accordingly; all changes are highlighted in red in the manuscript (main text, figure legends, tables, references). The delay for submitting this major revision is explained by the time needed to perform and analyze new experiments, particularly those in which we investigated the effect of β2/β3 agonists upon inhibition of PKA by H89.

We have added one co-author, Bartosz Wasicki, who contributed to analyze the new series of experiments where we used the H89 PKA inhibitor, whose results are displayed in a new Supplemental Figure (Supplemental Figure 1). Below we provide a point-by-point reply to the reviewers´ comments.

We look forward to hearing from you, hopefully for positive feedback.

Guillaume Caron, Daniel Zytnicki, Marcin Baczyk and Francesco Roselli

Reviewer comments:

Reviewer #1: In this paper, the authors investigated homeostatic feedback mechanisms regulating motoneuron excitability in presymptomatic SOD1-G93A ALS mice. They found that β2 and β3 adrenergic receptors are expressed in spinal motor neurons of both WT and SOD1 mice at P45 and play a role in neuromodulation. Activation of these receptors influences motoneurons firing properties through ion channel transcriptional changes, leading to receptor downregulation as part of a homeostatic response. Importantly, they report that homeostatic feedback loops remain intact in presymptomatic SOD1 mice, suggesting that early-stage ALS does not disrupt these regulatory mechanisms.

This study provides valuable insights into adrenergic modulation of motoneuron excitability in presymptomatic ALS by contributing to the understanding of homeostatic mechanisms in early ALS pathology. The study is well-structured, with clear experimental design, making a significant contribution to the field of ALS research.

>>>>We thank the reviewer for his/her positive comments on our work.

Major suggestions:

- ALS is a progressive neurodegenerative disease, and the presymptomatic phase may exhibit subtle changes that are not immediately detectable at P45. I would suggest that the authors consider additional time points beyond P45 to assess whether homeostatic feedback remains intact throughout the presymptomatic phase. It is possible that subtle dysregulations in homeostatic mechanisms may not be visible at P45 but could emerge later in the disease course, even before the onset of motor symptoms. Including earlier (e.g., P30) or later (e.g., P60) time points might provide a more comprehensive view of the dynamics of feedback regulation in SOD1 mice and strengthen the conclusions about the stability of homeostatic mechanisms.

>>>>We appreciate the reviewer's suggestion. However we feel that P45 is the most important time point to look at. Actually, it is close to the denervation onset of the neuromuscular junctions (Pun et al., 2006). If the dysregulation of the homeostasis were to be a crucial driver of the degeneration process, it should be maximal at this particular time point. Later on, after P60, the NMJs of the most vulnerable motor units have already degenerated, although these degeneration do not yet translate into substantial motor symptoms. Investigating such a late time point would then not allow us to pinpoint the pathophysiological mechanisms that may cause the degeneration of NMJs. Moreover, performing our vivo experiments at a young age such as P30 is at the limit of the feasibility. Indeed, performing intracellular recordings of spinal motoneurons is technically challenging and demanding in mice. Each experiment (a single mouse) lasts a full day and we can only successfully record a small number of motoneurons in a single experiment. Given the number of conditions (WT vs mSOD1 mice; acute vs chronic; vehicle vs beta-adrenergic agonists) we anticipated more than one year of work for investigating a single additional time point which is not a realistic time schedule for a resubmission.

Pun S, Santos AF, Saxena S, Xu L, Caroni P. Selective vulnerability and pruning of phasic motoneuron axons in motoneuron disease alleviated by CNTF. Nat Neurosci. 2006 Mar;9(3):408-19. doi: 10.1038/nn1653.

-While assessing misfolded SOD1 levels is valuable, it may not be sufficient to conclusively claim no impact on motoneurons. Other measures of motoneuron integrity should be considered, such as for instance motoneurons count (e.g., ChAT, NeuN) and axonal degeneration assessment (e.g. NFL, SMI32) in spinal cord sections.

>>>> We have now integrated the analysis of the impact of the beta-adrenergic agonists including, besides misfolded SOD1 burden, the total number of MN identified by cytoplasmic VAChT immunoreactivity in a representative set of spinal cord sections obtained from lumbar spinal cord, and the immunofluorescence intensity of the autophagy marker p62 (which accumulates upon autophagy overload). Both the MN number and the p62 load revealed no change upon chronic beta-agonists treatments. These additional results are reported in the lines 483-489 and in Fig. 8E-G (and corresponding legend).

- I would suggest that the authors consider expanding the investigation to include the effects of β2 adrenergic receptor antagonists on motoneuron excitability, which could provide valuable insights into the mechanistic role of β2 receptors in ALS pathology.

>>>> The experiment proposed by Reviewer is indeed of interest, given the broad use of beta-blockers in clinical therapeutics. The execution of the experiment is complicated in the electrophysiological setting by the use of Medetomidine, an alpha2-adrenergic agonist, in the anesthesia maintenance. Since alpha2-agonists reduce adrenergic synapses neurotransmitter release, the baseline adrenergic tone is anticipated to be already low and may be difficult to detect further decreases in adrenergic signaling by blocking beta-receptors. Furthermore, Atropine is administered to the animals to stabilize their heart rate during the procedures, a goal that could be harder to achieve under beta-blockers administration. In fact, the beta-blocker experiment could only be performed under chronic administration conditions and in vehicle vs treated design to observe the long-term effects; however, being unable to test the acute vs the chronic effects, it would be impossible to identify the effects of adaptation and desensitization vs the effects of the blockers themselves. The effect of beta-blockers could be deduced only indirectly using transcriptional or histological markers, with an experimental design that may stray away from the focus of the current paper. We have now mentioned this possible outlook in the discussion paragraph dedicated to the limitations of the study (lines 671-677).

-If homeostatic feedback remains intact at P45, how do the authors explain this finding with the progressive motoneuron dysfunction observed in ALS mice? Would disrupting these adrenergic feedback loops accelerate or slow disease progression? A discussion on the potential therapeutic implications of β-adrenergic modulation would strengthen the manuscript.

>>>>Actually our data demonstrate that the homeostasis of the beta-adrenergic neuromodulation of motoneuron excitability is not dysregulated in ALS mice at the onset of neuromuscular junctions degeneration of the most vulnerable motor units. This conclusion suggests that beta-adrenergic neuromodulation has most likely no effect on the early pathophysiological mechanisms of ALS. We now expand the Introduction (lines 131-134) and the Conclusion paragraph (lines 701-705) to make this issue clearer.

Reviewer #2: The study aims to investigate whether neuromodulation of motor neuron (MN) intrinsic properties is homeostatically regulated in presymptomatic adult SOD1 ALS mice compared to age-matched wild-type (WT) controls.

Specifically, the researchers examine the expression and regulation of Gs-coupled β2- and β3-adrenergic receptors in MNs, assessing their role in modulating MN firing through the cAMP/PKA pathway. By analyzing receptor desensitization following chronic activation, the study seeks to determine whether homeostatic plasticity of β-adrenergic signaling occurs in ALS MNs compared to WT controls and whether this mechanism contributes to MN excitability regulation, potentially identifying therapeutic targets.

Although this is an original and innovative topic, a key concern is the limited evidence linking these findings to MN neuropathology and ALS disease.

>>>>We thank the reviewer for his/her positive comments. Actually our study demonstrates that the homeostatic plasticity of the beta-adrenergic signaling is not dysregulated in ALS MNs compared to WT controls around P45, i.e. at the denervation onset of the most vulnerable motor units. This finding suggests that the beta-adrenergic neuromodulation has most likely no effect on the early pathophysiological mechanisms of ALS. We now expand the Introduction (lines 131-134) and the Conclusion paragraph (lines 701-705) to make this issue clearer.

Specific Comments:

1-The selective increase of Adrb1 mRNA exclusively in vulnerable (Mmp9+) MNs at the early presymptomatic stage suggests that β1-adrenergic receptors may play a crucial role in ALS pathogenesis. However, the study focuses solely on Adrb2/3, which remain unchanged and are assumed to contribute to the homeostatic regulation of MN intrinsic properties. This conceptual framework is a bit difficult to grasp and, in my opinion, deserves a clearer justification.

>>>> We agree on the potential relevance of Adrb1 as an indicator or as a contributor of the pathogenic pathway affecting vulnerable MN, demonstrated by the expression data. However, including Adrb1 among the receptor panel studied to reveal their homeostatic response was not feasible for several reasons: i) Adrb1 agonists (e.g., isoproterenol, xamoterol) do not cross the blood-brain-barrier in substantial quantities due to their polarity: for xamoterol, the brain penetration is in the low- ng/g range (Yi et al., 2017); ii) at the very high doses necessary to obtain any degree of brain penetration, Adrb1 agonists are anticipated to have substantial cardiovascular and metabolic effects, confusing the interpretation of the results and possibly interfering with the well being or survival of the animals; iii) a newly identified Adrb1 agonist (SD-101) with improved brain permeability also displays strong affinity for multiple other GPCR (including 5-HT1A, 5-HT2B, α1A adrenergic, α1D adrenergic, and D3 dopaminergic receptors; Supplementary Table 1 contained in Yi et al., 2017), making it unsuitable for the selective activation of Adrb1. Thus, being the simultaneous stimulation of all three Adrb receptors not feasible with suitable selectivity, we opted to target only Adrb2 and Adrb3 (for which small molecules with suitable pharmacokinetics and selectivity do exist), which were not downregulated and therefore available for stimulation. We have now clarified the framework of limitations in the results chapter (lines 167-174).

2-In Figure 2, the authors demonstrate that acute intraperitoneal (IP) injection of β2 and β3 agonists (AF) activates PKA signaling (phosphorylation) in SOD1 MNs but not in WT MNs three hours post-treatment. However, the same treatment induces immediate-early gene expression (cFos, FosB) in laser-captured MNs (LCM-MNs) of both WT and SOD1 mice, suggesting that these two events (PKA activation and altered gene expression) may not be strictly linked.

>>>> The interpretation of the apparent divergence of Fig. 2A and 2B should take into consideration that the sensitivity of the two techniques (immunostaining vs qPCR) is different, with larger signal amplification (i.e., higher sensitivity) anticipated for the qPCR; furthermore, the effect size of phosphorylation changes vs induction of immediate early genes is not comparable, since baseline for phosphorylation is non-zero in vivo, whereas cFos expression in motoneurons can be extremely low. Importantly, the statistical significance in Fig. 2A (Veh vs AF in WT) is negated by a single AF animal (lowest black point in the WT-AF scatterplot) that did not respond; excluding this animal would result in a trend toward elevation of PKA activity. Since the exclusion of this single animal could not be justified by pre-specified quality control criteria, we opted to include instead of altering the results. When the differences in sensitivity between techniques and the 4 out of 5 trends in Fig. 2A are taken into consideration, we believe that a substantial agreement still remains between signaling and immediate early genes induction experiments, and evidence of a decoupling between the two is a least-preferred option. We have clarified our reasoning in the results chapter (lines 203-210).

3-The electrophysiological analysis of MNs indicates that intravenous (IV) injection (not IP as above) of β2/β3 agonists enhances MN excitability by decreasing H-current in SOD1 MNs but not in WT MNs. Conversely, the treatment lowers the voltage threshold for firing in MNs of WT mice but not in SOD1 MNs. This suggests that ALS MNs do not fully

respond to β2/β3 adrenergic signaling. Could this be due to the lower basal levels of PKA signalling in SOD1MNs compared to WT MNs, making them less responsive? This limitation raises concerns about the efficacy of β2/β3 agonists in modulating SOD1 MNs excitability.
Additionally, the rationale for using IV instead of IP administration should be clarified.

>>>> WT and SOD1 react differently to B2/B3 agonists since Gin Plateau/Gin Ramp decreases in SOD1 MNs but not in WT MNs whereas the voltage threshold for firing is lowered in WT MNs but not in SOD1 MNs. However, both WT and SOD1 exhibited an increase in the FI gain. These properties are mediated by different ion channels. It is possible that the sensitivity to PKA activation differs among these channels, and the lower basal levels of PKA activity in SOD1 might potentially explain the observed differences. Moreover, other signaling mechanisms, in particular beta-arrestin/Erk dependent and cAMP/non-PKA dependent, may also contribute to the differential effect of B2/B3 agonists on ion channels function. This is now stated in a new paragraph (3.4 Study limitations) added to the discussion (lines 677-685).

Repeated IV injections were not considered ethically feasible due to the burden on the mice, and using IP injections in the electrophysiological experiment would have negated the precise timing of the adrenergic activation, due to the broader kinetics proper of IP vs IV. Indeed, although we could not locate dataset providing a direct comparison for amibegron and/or formoterol IP vs IV pharmacokinetics, extrapolating from the pharmacokinetics literature, we may assuage that IV may have generated a faster receptor saturation and faster inactivation than the IP. This faster receptor saturation allowed us to precisely determine the beginning of B2/B3 agonists effects on MN excitability. We provide a description of this limitation in the discussion chapter (lines 655-671).

4-To further evaluate whether the cAMP/PKA pathway mediates these effects, the authors intracellularly injected a cAMP analog into MNs and measured its impact on excitability. While this intervention mimics some effects of β2/β3 agonists, a more direct demonstration would require testing the effect of PKA inhibitors. Additionally, the authors did not assess beta-arrestin/ERK activation, leaving open the possibility of an alternative signaling pathway involvement. Therefore, their conclusions regarding cAMP/PKA signaling should be tempered accordingly.

>>>> We thank the Reviewer for his/her suggestions to verify that PKA inhibitors abolished the beta adrenergic neuromodulation. We now performed a new series of in vivo experiments to determine whether the effects of Adrb2/Adrb3 agonists on the firing gain are abolished when PKA is blocked with H89. We compared the impact of Adrb2/Adrb3 agonists on the F-I gain in the absence or in the presence of H89. In the absence of H89, we found again that Adrb2/Adrb3 agonists increase the firing gain but the firing gain was not increased anymore in the presence of H89, confirming that PKA is involved in the beta-adrenergic neuromodulation of the firing gain. The results of these experiments are shown in a new figure (Supplemental Figure 1 and corresponding legend) and described in the results section 2.4 (lines 348-356). Finally, we also acknowledged the existence of other signaling mechanisms, in particular beta-arrestin/Erk dependent and cAMP/non-PKA dependent, which may contribute to the Adrb2-3 impact on excitability and ion channels function. We added a corresponding paragraph to the discussion (lines 677-685)

Direct cAMP/PKA activation in MNs was also achieved using DREADD expression via AAV9 in SOD1/ChAT-Cre double-transgenic mice (Figure 8). Why the authors did not monitor these mice electrophysiologically? It would have been a validation and support of previous observations.

>>>> We agree with the Reviewer that this experiment would have been most interesting. However, this requires the high-efficiency genetic access to an identified population of MN in the spinal cord, excluding the expression in other neurons or cell types, so that the random and blind intracellular recordings of motoneurons would hit a substantial fraction of MN expressing the DREADD. This feat is currently not possible. Large-scale infection of spinal cord MN, excluding interneurons, cannot be achieved with intrathecal or intravenous injection of AAVs, and genetic access to Cholinergic cells in the spinal cord affects only a fraction of MNs (our blind intracellular recordings would not allow to specifically target the MN expressing the DREADD). Thus, the experiment remains to be performed once suitable tools become available.

The study reports that a single dose of β2/β3 agonists significantly alters ion channel gene expression within three hours in both WT and SOD1 mice. If the treatment was administered via IP, the pharmacokinetics of IP versus IV routes should be discussed, as they could differentially impact MN excitability.

The prolonged delivery of β2/β3 agonists, administered via IP, was compared to the acute IV treatment. This may be a critical issue when comparing the responses. It is unclear whether the second experiment conducted by an independent group in another lab was done under the same conditions. This should be clarified.

>>>> We agree with the Reviewer that different administration routes, IP vs IV, may affect peak serum concentration and time-to-peak, although it affects much less the overall Area-Under the-Curve measures (AUC; see for example Yi et al. 2017; al-Shoyab et al., 2020). We could not locate dataset providing a direct comparison for amibegron and/or formoterol IP vs IV pharmacokinetics, but, extrapolating from the pharmacokinetics literature, we may assuage that IV may have generated a faster receptor saturation and faster inactivation than the IP. At any rate, repeated IV injections were not considered ethically feasible due to the burden on the mice, and using IP injections in the electrophysiological experiment would have negated the precise timing of the adrenergic activation, due to the broader kinetics proper of IP vs IV. We provide a description of this limitation in the discussion chapter (lines 655-671). We further clarify that the second experiment, conducted by an independent group in another lab, followed exactly the same protocol of IP/IV injection, mouse strain, anesthesia, and intracellular recording window (lines 458-459).

Chronic β2/β3 agonist administration reduced β1-β3 receptor and Drd5 mRNA levels, whereas chronic chemogenetic PKA activation selectively reduced β1 mRNA. This discrepancy suggests the involvement of additional pathways in the long-term effects of β2/β3 receptor activation, which warrants further investigation.

>>>> The DREADD-Gs was originally derived from a muscarinic receptor in which the G-protein-coupling loops were swapped with those of the turkey adrenergic beta-1 receptor. Therefore, the DREADD recapitulates the Gs signaling but may not fully recapitulate other signaling properties; the high level of expression, the lack of negative feedback and the non-localized membrane insertion may also contribute to mimicking the signaling landscape of endogenous receptors. The experiment was performed to verify if Gs signaling activated by DREADD-Gs, instead of a native adrenergic receptor, was sufficient to simulate chronic adrenergic stimulations; the outcome of the experiment remains compatible with a large overlap between DREADD-Gs and Adrb2/b3 stimulation. The possible divergences are now briefly mentioned in the discussion chapter (lines 685-691).

Conclusion:
This study provides valuable insights into the modulation of MN excitability via β2/β3 adrenergic receptors and contributes to understanding the homeostatic feedback mechanisms involved. However, the absence of pathological and functional outcomes related to ALS, such as MN loss, morphological /histopathological MN damage, or even neuroinflammation reactivity in spinal cord, raises questions regarding the therapeutic relevance of these findings. Addressing these limitations would strengthen the study's impact and translational potential.

Reviewer #3: This is a groundbreaking study that demonstrates for the first time neuromodulation through beta2/3 adrenergic receptors impacts MN transcription and translation, including altered expression of voltage gated ion channels, dopaminergic and adrenergic receptors in the receptive MNs. This is novel for spinal MNs, as previous work has documented adrenergic effects in MNs via alpha and beta1 receptor activation. The authors further demonstrate that presymptomatic SOD1 MNs respond largely in a similar way to beta2/3 modulation as WT MNs.

This paper presents a lot of great results, but it is not quite organized in a way that makes sense. There seems to be a disconnect between what is anticipated to be a paper on mechanisms of pathology in ALS and what turns out to be more of a paper on regulation of neural excitability of MNs in a more general sense. It might benefit the authors to decide what the main thrust of this paper is. If it is the difference between WT and SOD1 MNs, then pursuing more complete results on the effects of the increased expression of beta1 adrenergic receptors in SOD1 MNs would be essential. What is the impact of the increased expression, if indeed the increase in adrb1 mRNA results in increased surface expression of the receptors, and increased sensitivity to beta1 agonists in SOD1 MNs. Authors state that "We de-prioritized the β1 receptor because it was already affected by the disease, agonists do not cross the blood-brain barrier well and they produce substantial systemic effects." De-prioritizing the beta1 receptor because it is affected by the disease does not really make sense if the paper's focus is on disease mechanisms of ALS. It would possibly make sense if the receptor expression appeared to be down regulated in SOD1 MNs and they would likely be less sensitive to receptor activation but in fact they are likely more sensitive, not less. Alternatively, this could also be a great paper focused on a novel pathway for neuromodulation of MNs via beta2/3 receptor activation.

>>>> We thank the reviewer for his/her positive comments on our work. We agree on the potential relevance of Adrb1 as an indicator or as a contributor of the pathogenic pathway affecting vulnerable MN, demonstrated by the expression data. However, including Adrb1 among the receptor panel studied to reveal their homeostatic response was not feasible for several reasons: i) Adrb1 agonists (e.g., isoproterenol, xamoterol) do not cross the blood-brain-barrier in substantial quantities due to their polarity: for xamoterol, the brain penetration is in the low- ng/g range (Yi et al., 2017); ii) at the very high doses necessary to obtain any degree of brain penetration, Adrb1 agonists are anticipated to have substantial cardiovascular and metabolic effects, confusing the interpretation of the results and possibly interfering with the well being or survival of the animals; iii) a newly identified Adrb1 agonist (SD-101) with improved brain permeability also displays strong affinity for multiple other GPCR (including 5-HT1A, 5-HT2B, α1A adrenergic, α1D adrenergic, and D3 dopaminergic receptors; in Yi et al., 2017,), making it unsuitable for the selective activation of Adrb1. Thus, being the simultaneous stimulation of all three Adrb receptors not feasible with suitable selectivity, we opted to target only Adrb2 and Adrb3 (for which small molecules with suitable pharmacokinetics and selectivity do exist), which were not downregulated and therefore available for stimulation. We have now clarified the framework of limitations in the results chapter (lines 167-174).

AHP measurements taken from firing on current ramps. Authors cite Manuel et al 2006 for the measurements, but the 2006 study used APs evoked by short stimuli delivered at 1-2Hz, while the present study averaged spikes from the sub primary zone of spikes fired in response to a current ramp. Based on the example in figure 4H-I the firing rate was ~15 Hz, which seems bit too fast to allow for an analysis of the AHP.
There was a decrease in the plateau conductance of SOD1 (but not WT) MNs in the presence of AF drugs to activate beta2/3 receptors. However, it appears that the "group" effect was not significant. Is that correct? Were persistent inward currents decreased by beta2/3 adrenergic receptor activation? Was conductance different between WT and SOD1 MNs?
How were the impacts of CNO on dopaminergic receptors controlled for in the DREADDs experiment?

>>>>We estimated the AHP half decay time on spike triggered averaging of the first 3-5 spikes in the subprimary range, as in Figure 4G and 4I. We verified that in the selected interspikes intervals the AHP fully relaxed; the half decay time was on average 4.5 times shorter than the average of interspike intervals. In the example shown in Figure 4G, the 3 first interspike intervals were 83, 58 and 59 ms, respectively. On the spike triggered averaging of the first 3 spikes (Fig. 4I), an AHP is clearly visible with a half relaxation of 9.6 ms, i.e. at least 6 times shorter than the interspike intervals, which allows the AHP to fully relax. With this method, the distribution and mean of the AHP half-decay time before AF injections are from 5.8 ms to 14 ms (average of 9.7 ms; SD +/- 2.2 ms) which are very similar from the values found in Manuel et al. (2009). These precisions are reported in the legend of Figure 4 and we expanded the material and methods (section 5.3) to explain how we performed the measurements (lines 794-800).

In the case of plateau conductance, the p value for the interaction effect is equal to 0.057, i.e. a value very close to the significance threshold. Now, for the sake of clarity, we opted for writing the exact value numbers in all figure panels.

To demonstrate whether beta adrenergic receptor activation modulates persistent inward currents, we would have had to perform voltage clamp experiments during triangular ramps of voltage in addition to the voltage clamp recordings required to investigate the firing properties of MNs. We opt to not perform such difficult experiments because switching for each MN from current clamp recordings to voltage clamp recordings would require to keep good quality intracellular MN recordings for a long time (maybe 30 minutes or more) which is hard to achieve in vivo in mice. In addition, given the longer time to investigate each MN, the number of recorded MNs per experiment would have been significantly reduced.

Clozapine-n-oxide can be retro-metabolized into Clozapine, which displays antagonist activity for the D4/D1, serotonin 5-HT2A, and alpha-adrenergic receptors (Reynolds, 2025) and possible effects on the receptors include compensatory upregulation (Giorgi et al., 1993) or downregulation (Lidow et al., 1997). In our experiment, we considered from the same animal motoneurons expressing the DREADD-Gs or not from the same sections, so that all cells were exposed to the same concentration of CNO. Since CNO was injected systemically, the non-DREADD-dependent pharmacological effects were exerted equally on all cells, DREADD positive or negative, but the DREADD-specific effects only appeared in the cells expressing DREADD. We have clarified this point in the Results chapter (lines 472-476).

Giorgi O, Pibiri MG, Loi R, Corda MG. Chronic treatment with SCH 23390 increases the production rate of dopamine D1 receptors in the nigro-striatal system of the rat. Eur J Pharmacol. 1993 Apr 15;245(2):139-45. doi: 10.1016/0922-4106(93)90121-o.

Yi B, Jahangir A, Evans AK, Briggs D, Ravina K, Ernest J, Farimani AB, Sun W, Rajadas J, Green M, Feinberg EN, Pande VS, Shamloo M. Discovery of novel brain permeable and G protein-biased beta-1 adrenergic receptor partial agonists for the treatment of neurocognitive disorders. PLoS One. 2017 Jul 26;12(7):e0180319. doi: 10.1371/journal.pone.0180319.

Lidow MS, Elsworth JD, Goldman-Rakic PS. Down-regulation of the D1 and D5 dopamine receptors in the primate prefrontal cortex by chronic treatment with antipsychotic drugs. J Pharmacol Exp Ther. 1997 Apr;281(1):597-603.

Silvestri S, Seeman MV, Negrete JC, Houle S, Shammi CM, Remington GJ, Kapur S, Zipursky RB, Wilson AA, Christensen BK, Seeman P. Increased dopamine D2 receptor binding after long-term treatment with antipsychotics in humans: a clinical PET study. Psychopharmacology (Berl). 2000 Oct;152(2):174-80. doi: 10.1007/s002130000532.

Assessment of receptor expression. It not entirely clear if there is altered expression of beta adrenergic receptors in WT vs presymptomatic SOD1 MNs – transcriptomics indicates upregulation of Adrb1, downregulation of Adrb2 type beta adrenergic receptors, and no change in Adrb3 receptors, however in situ hybridization only supports the increase in Adrb1 / beta1 adrenergic receptor expression. Note that for Adrb1, where it appears there were 6-7 mice per group, results were significant, and non-sig for the other receptor subtypes where there were only 4 animals per group. It's unfortunate that a specific assessment of the surface expression of the receptor proteins was not possible.

*>>>> In the comparison of the Fig. 1A and 1B, the following pattern can be detected: Adrb1, **upregulated** in transcriptome, **upregulated** in vulnerable MN, unchanged in non-vulnerable MN; Adrb2, **downregulated** in transcriptome, unchanged in vulnerable MN, **tendency toward downregulation** in non-vulnerable MN; Adrb3, **unchanged** in transcriptome, **unchanged** in vulnerable MN, **trend toward a decrease (of very small degree)** in non-vulnerable MN. Thus, the biological effects are compatible with the only exception of Adrb2-vulnerable MN: in this case, three of four animals display a substantial downregulation, with one generating the opposite result. If the statistical analysis is performed MN-wise instead of animal-wise, a significant downregulation is observed. To avoid pseudoreplication and biasing of results by unduly exclusion of animals, we kept the analysis animal-wise and we did not exclude any animal based on outcomes. When considering the natural variability, the different sensitivity of the two techniques and the microdissection focus on large MN, we believe that the results are showing similar overall trends. These findings are highlighted in the results chapter (lines 160-166).*

The difference between the number of animals in Adrb1 vs Adrb2/3 is due to the execution of the experiments: since single-molecule in situ is logistically cumbersome, the samples were divided in two sets. To assure a similar set of conditions, one receptor was duplicated (Adrb1 and Adrb2 in one set, and Adrb1 and Adrb3 in the second set). To avoid any selection bias in the data presented (i.e., the selection of one of the two Adrb1 datasets), we displayed them together, since both pointed to the same result (upregulation in Mmp+) and both agreed with the transcriptome from microdissected MN. We have clarified this point in the methods (lines 920-926).

We considered the possibility to use immunostaining to determine the actual expression of adrenergic receptors at protein level, and to characterize their localization. However, testing of several commercial antibodies against wt and adrb3 or adrb2-KO samples revealed that most, if not all, of the immunoreactivity was equally present in the wt and ko samples, pointing toward lack of specificity (this problem has been previously raised: Michel MC, Wieland T, Tsujimoto G. How reliable are G-protein-coupled receptor antibodies? Naunyn Schmiedebergs Arch Pharmacol. 2009 Apr;379(4):385-8. doi: 10.1007/s00210-009-0395-y).

Language is unclear. For example, authors refer to beta2 and beta3 adrenergic receptors in the introduction but don't mention which adrenergic receptors are encoded by which gene. The authors then switch to gene names in results section and it's not clear to readers which one is being studied. Further, rather than saying "acute" for the short term application of FA, it is referred to as "punctual," which means "on time" and does not make sense in this context. In section 2.5, 19 ion channel genes are listed. It would be very helpful to also list which channels those genes encode.

>>>> We have modified the language so that standard gene/mrna transcript names are indicated in italics and the same standard names are used non-italicized to indicate proteins. For the ion channels, an additional table column has been added to indicate the conventional names along with the standard gene names in the supplementary table that includes the primer sequences..
The word "punctual" has been removed and substituted with "acute" or "single-dose" as appropriate.

Immediate early genes. In every gene assessed, the vehicle-treated SOD1MNs look similar to the AF-treated WT MNs, as if the immediate early genes are already activated in the SOD1 MNs. Is this a known early indicator of disease related cell stress? In contrast, why would the RRxpS/T levels be lower than normal?

>>>> As the Reviewer pointed out, immediate-early genes, prototypically represented by c-fos, not only respond to neuronal activity and activity-related signaling, but are also induced by other types of stimuli. In particular, fos was shown to be induced by oxidative stress (Rao et al., 1993 ;Yatin et al., 2002), heat shock (Sharp and Sagar, 1994), ischemia (Liverman et al., 2004) and inflammatory stimuli (Elmquist et al., 1993; Leite et al., 2016). Therefore, the baseline elevation may be due to the ongoing pathogenic process, which is known to involve oxidative and inflammatory components. The upregulation is strictly related to the intervention (i.e., the agonist administration), and therefore is a readout of the cellular responses.

On the other hand, the phosphorylation of the RRxpS/T motif reflects the degree of PKA signaling in the cell; PKA is itself activated by synaptic activity, and downregulation of glutamatergic receptors at excitatory synapses (as shown in Baczyk et al., 2020) may be sufficient to account for the lower baseline.

Brief clarifications of both points have been added to the results chapter (lines 203-204 and 214-218).

Elmquist JK, Ackermann MR, Register KB, Rimler RB, Ross LR, Jacobson CD. Induction of Fos-like immunoreactivity in the rat brain following Pasteurella multocida endotoxin administration. Endocrinology. 1993 Dec;133(6):3054-7. doi: 10.1210/endo.133.6.8243337. PMID: 8243337.

Yatin SM, Miller GM, Norton C, Madras BK. Dopamine transporter-dependent induction of C-Fos in HEK cells. Synapse. 2002 Jul;45(1):52-65. doi: 10.1002/syn.10084. PMID: 12112414.

Leite HR, Oliveira-Lima OC, Pereira LM, Oliveira VEM, Prado VF, Prado MAM, Pereira GS, Massensini AR. Vesicular acetylcholine transporter knock down-mice are more susceptible to inflammation, c-Fos expression and sickness behavior induced by lipopolysaccharide. Brain Behav Immun. 2016 Oct;57:282-292. doi: 10.1016/j.bbi.2016.05.005. Epub 2016 May 11. PMID: 27179819.

Liverman CS, Cui L, Yong C, Choudhuri R, Klein RM, Welch KM, Berman NE. Response of the brain to oligemia: gene expression, c-Fos, and Nrf2 localization. Brain Res Mol Brain Res. 2004 Jul 5;126(1):57-66. doi: 10.1016/j.molbrainres.2004.02.028. PMID: 15207916.

Rao GN, Lasségue B, Griendling KK, Alexander RW, Berk BC. Hydrogen peroxide-induced c-fos expression is mediated by arachidonic acid release: role of protein kinase C. Nucleic Acids Res. 1993 Mar 11;21(5):1259-63. doi: 10.1093/nar/21.5.1259. PMID: 8464709; PMCID: PMC309291.

Sharp FR, Sagar SM. Alterations in gene expression as an index of neuronal injury: heat shock and the immediate early gene response. Neurotoxicology. 1994 Spring;15(1):51-9. PMID: 8090362.

Proofreading is needed throughout the manuscript

Line 272 mentions “full blown spikes” but are spikes not consistently all-or-none?

>>>>We wrote “full blown spikes” to make an opposition with what happens in the subprimary range in which oscillations are present between the spikes (see Iglesias et al 2011). For the sake of simplicity we now deleted the words “full-blown spikes”. The sentence now read: “...., before transitioning to a regime of firing where spike frequency increases linearly with the injected current, i.e. the primary range.” (now lines 282-284).

Labels for significance are confusing, including both asterisks and hashtags above each bracket. Please clarify when interaction is found, what variables they are referring to.

>>>>For the sake of clarity, we now opted to delete any reference to “treatment effect across genotypes” in the figures and in the text (however we kept the corresponding p values in the Supplemental Excel table reporting all the statistics). We now wrote the exact p values for the interaction effects in all figure panels.

Figure 4B Voltage acceleration would be easier to see if a straight line was added, at the initial slope of the voltage increase.

>>>> We have modified the figure to add a straight line at the initial slope of the voltage increase.

Fig 4G Arrows are not really effective at showing the variability in firing rate - including the frequency plot above would be helpful here.

>>>> We have modified the figure to add a frequency plot.

Legend for Figure 4 seems to list the panels out of sequential order

>>>> Legend has been amended to remove this mistake, we apologize about this.

2nd Decision Letter

Dear Dr. Caron,

Thank you for submitting your manuscript to Progress in Neurobiology.

We have completed our evaluation of your manuscript. The reviewers recommend reconsideration of your manuscript following major revision. We invite you to resubmit your manuscript after addressing the comments below. Please resubmit your revised manuscript by **Mar 04, 2026**.

When revising your manuscript, please consider all issues mentioned in the reviewers' comments carefully: please outline every change made in response to their comments and provide suitable rebuttals for any comments not addressed. Please note that your revised submission may need to be re-reviewed.

To submit your revised manuscript, please log in as an author at https://www.editorialmanager.com/proneu/, and navigate to the "Submissions Needing Revision" folder.

Research Elements (optional)
This journal encourages you to share research objects - including your raw data, methods, protocols, software, hardware and more – which support your original research article in a Research Elements journal. Research Elements are open access, multidisciplinary, peer-reviewed journals which make the objects associated with your research more discoverable, trustworthy and promote replicability and reproducibility. As open access journals, there may be an Article Publishing Charge if your paper is accepted for publication. Find out more about the Research Elements journals at https://www.elsevier.com/authors/tools-and-resources/research-elements-journals?dgcid=ec_em_research_elements_email.

Progress in Neurobiology values your contribution and we look forward to receiving your revised manuscript.

Kind regards,

Jeannie Chin, PhD
Co-Editor-in-Chief
Progress in Neurobiology

Editor and Reviewer comments:

Editorial: Please consider the comments of Reviewer 3 regarding the interpretability of the AHP analysis, and either remove from the manuscript as suggested or thoroughly address the concern raised.

Reviewer 1: (No further comments were received)

Reviewer 3: This is a solid paper that contains interesting data and it would benefit the field for it to be published, but the authors were only somewhat responsive to comments. Unfortunately, for many reasons including limited resources and time, it is typical that a few suggestions may be deferred to another study, but it seemed that more issues were brushed aside than addressed. For example, their response on the AHP analysis was unsatisfactory.

Major issues
AHP Analysis and firing rate: The authors countered my previous comment by saying that "On the spike triggered averaging of the first 3 spikes (Fig. 4I), an AHP is clearly visible with a half relaxation of 9.6 ms, i.e. at least 6 times shorter than the interspike intervals, which allows the AHP to fully relax. With

this method, the distribution and mean of the AHP half-decay time before AF injections are from 5.8 ms to 14 ms (average of 9.7 ms; SD +/- 2.2 ms) which are very similar from the values found in Manuel et al. (2009). These precisions are reported in the legend of Figure 4 and we expanded the material and methods (section 5.3) to explain how we performed the measurements (lines 794-800)."

Again, based on the Manuel et al 2009 paper, the total AHP duration is actually 6 times longer than the AHP1/2 decay time, so it seems the end of the AHP and the beginning of the next spike are co-occurring, making it more difficult to interpret. I do not think the AHP analysis should be included.

Minor issues
Results section 2.3 Specify for readers which MNs were targeted here. Was the intention to sample MN of different firing types but from just from one motor pool for consistency?

2nd Author Response Letter

Editor and Reviewer comments:

Editorial: Please consider the comments of Reviewer 3 regarding the interpretability of the AHP analysis, and either remove from the manuscript as suggested or thoroughly address the concern raised.

The reviewer comments made us realize that our explanations were not clear enough regarding the interpretability of the AHP analysis. We opted to thoroughly address the concern raised (page 12 in the 2.3 section; page 16 in the legend of Figure 4; page 26 in the 3.2 section and page 34 in the 5.3 section). We hope that our explanations are now clearer. We would prefer to keep the AHP analysis because we believe that the acceleration of its kinetics by the Adrb2/Adrb3 agonists may mechanistically contribute to increasing the F-I gain in the primary firing range.

Note that all changes in the text are highlighted in green (changes in red were made in the previous revisions).

Reviewer 1: (No further comments were received)

Reviewer 3: This is a solid paper that contains interesting data and it would benefit the field for it to be published, but the authors were only somewhat responsive to comments. Unfortunately, for many reasons including limited resources and time, it is typical that a few suggestions may be deferred to another study, but it seemed that more issues were brushed aside than addressed. For example, their response on the AHP analysis was unsatisfactory.

Major issues

AHP Analysis and firing rate: The authors countered my previous comment by saying that "On the spike triggered averaging of the first 3 spikes (Fig. 4I), an AHP is clearly visible with a half relaxation of 9.6 ms, i.e. at least 6 times shorter than the interspike intervals, which allows the AHP to fully relax. With this method, the distribution and mean of the AHP half-decay time before AF injections are from 5.8 ms to 14 ms (average of 9.7 ms; SD +/- 2.2 ms) which are very similar from the values found in Manuel et al. (2009). These precisions are reported in the legend of Figure 4 and we expanded the material and methods (section 5.3) to explain how we performed the measurements (lines 794-800)."

Again, based on the Manuel et al 2009 paper, the total AHP duration is actually 6 times longer than the AHP1/2 decay time, so it seems the end of the AHP and the beginning of the next spike are co-occurring, making it more difficult to interpret. I do not think the AHP analysis should be included.

We would like to thank the reviewer whose comments made us realize that our explanations were not clear enough regarding the interpretability of the AHP analysis. We opted to

thoroughly address the concern raised (page 12 in the 2.3 section; page 16 in the legend of Figure 4; page 26 in the 3.2 section and page 34 in the 5.3 section).

We hope that our explanations are now clearer. Indeed, we would prefer to keep the AHP analysis because we believe that the acceleration of its kinetics by the Adrb2/Adrb3 agonists may mechanistically contribute to increasing the F-I gain.

We amended the text as following:

Page 12 in the 2.3 section (Results):

"It was established, both theoretically and experimentally, that the F-I gain in the primary firing range is proportional to the reciprocal of the AHP relaxation time constant (not of the AHP total duration): shorter the time constant, higher the gain (Kernell 1999, Meunier and Borejsza, 2005; Manuel *et al.*, 2006). In contrast, the firing frequency in the sub-primary firing range is not controlled by the AHP because the AHP has fully, or almost fully, relaxed before the next spike (Manuel et al. 2009, Iglesias et al. 2011). This feature makes it possible to measure the AHP half-decay time in the sub-primary range to estimate the possible changes in AHP kinetics. Of note, the AHP half-decay time is easier to measure than the AHP relaxation time constant (see Materials and Methods, section 5.3) but they are, on average, close to each other (see Table 2 in Manuel et al. 2009). We averaged the AHP following 3-5 spikes with the longest interspike intervals in the sub-primary range and we measured the AHP half-decay time on the averaged trace (see Materials and Methods, section 5.3; Fig. 4H). Interestingly, the treatment with Adrb2/Adrb3 agonists caused a significant shortening of the AHP half-decay time in WT MNs, and an almost significant shortening (p=0.07) in SOD1 MN (Fig. 4K). The treatment has then accelerated the AHP kinetics in the sub-primary range. It is likely that a similar acceleration of AHP kinetics took place also in the primary range, which may contribute to the observed increase in the F-I gain of the primary range."

Page 16 in the legend of Figure 4 (we reworded the description of panel F):

"*i.e.* at least 6 times shorter than the interspike intervals allowing the measurement of AHP half decay-time before the next spike."

Page 26 in the 3.2 section (Discussion):

"We found here that Adrb2/Adrb3 receptors agonists shortened the AHP half-decay time (a parameter close to the AHP time constant (Manuel et al. 2009)) in the sub-primary firing range. It is then very likely that the Adrb2/Adrb3 agonists also accelerated the AHP kinetics in the primary range, which may, at least partly, contribute to the F-I gain increase in this firing range."

Page 34 in the 5.3 section (Materials and Methods):

"The AHP half-decay time is the time between the peak amplitude and half the peak amplitude, with the peak being measured between the voltage before spike and the most

hyperpolarized voltage of the AHP. However, the AHP relaxation time constant is measured by fitting an exponential on the tail of the AHP, which is highly sensitive to the noise on the tail. Therefore, the AHP half decay time is easier to estimate using a 3-5 spike triggered average."

Minor issues

Results section 2.3 Specify for readers which MNs were targeted here. Was the intention to sample MN of different firing types but from just from one motor pool for consistency?

As data in Result sections 2.1 and 2.2 were obtained from random samples of MNs of the caudal part of the lumbar enlargement without distinguishing the motor pools, we randomly recorded, for the sake of consistency, MNs from hindlimb muscles innervated by the tibial and common peroneal nerves."

We have added the following clarification in the first paragraph of Results section 2.3:

"We randomly recorded MNs from hindlimb muscles innervated by the tibial and common peroneal nerves (Materials and Methods, section 5.3)."
